# On the Role of Food in the Transmission of *Helicobacter pylori* Infection: A Narrative Review

**DOI:** 10.3390/foods14244325

**Published:** 2025-12-15

**Authors:** Markus Schuppler

**Affiliations:** Institute of Food, Nutrition and Health, ETH Zurich, 8092 Zurich, Switzerland; markus.schuppler@ethz.ch

**Keywords:** *Helicobacter pylori*, infection, food, water, detection, reservoir, transmission, VBNC

## Abstract

*Helicobacter pylori* is one of the most common human pathogens, infecting up to 50% of the global population. The bacterium colonizes the mucus layer overlying gastric epithelial cells and causes chronic infection, which can lead to peptic ulcers, lymphoma, and gastric cancer. Epidemiological studies showed that regions with poor sanitation have higher prevalence rates of *H. pylori*, suggesting possible environmental or food-related transmission routes in addition to the well-established person-to-person pathways. This assumption is supported by the detection of *H. pylori* and/or its DNA in a variety of food. Experimental studies further demonstrate that *H. pylori* can survive in food with certain properties, such as milk, meat, and vegetables, suggesting that such products may serve as potential reservoirs. However, reliable detection of *H. pylori* in food remains challenging due to its fastidious nature, the ability to enter a viable but non-culturable state, and methodological limitations. While the presence of bacterial DNA and survival across food matrices make foodborne transmission biologically plausible, direct and conclusive proof that ingestion of contaminated food leads to infection is still lacking. Hence, person-to-person transmission currently remains the most firmly established route of transmission. Taken together, the current findings provide substantial indirect evidence that food, particularly under conditions of poor hygiene, may provide a reservoir or vehicle for *H. pylori* transmission. However, further research is needed to definitively clarify the role of food in the transmission of *H. pylori* infection and identify appropriate measures to promote public health.

## 1. Introduction

The genus *Helicobacter* comprises about 50 species that have been reported and isolated from stomach, gastrointestinal tract, liver, and gallbladder in more than 142 vertebrate species of mammals, birds, and reptiles [1]. The genus *Helicobacter* belongs to the Epsilon group of Proteobacteria and forms two groups: gastric *Helicobacter* species (GHS) and enterohepatic *Helicobacter* species (EHS), which differ in several aspects [2]. The tropism for different parts of the gastrointestinal system is also reflected in their phylogenetic relationship. Gastric species have been found to colonize the stomach of humans, dogs, cats, cheetahs, rhesus monkeys, ferrets, sheep, cattle, whales, and dolphins, while EHS are found in the liver, gall bladder or gastrointestinal tract of animals such as mice, rats, and hamsters [3,4]. Enterohepatic species may also colonize the lower gastrointestinal tract, including the biliary tree, in humans and other mammals. Persistent infections are often associated with chronic inflammation and hyperproliferation of epithelial cells, which can lead to neoplastic and hepatobiliary diseases in humans [5]. The species *Helicobacter pylori* belongs to the group of gastric *Helicobacter* species. All of them are microaerophilic, spiral-shaped bacteria showing a high motility due to a bundle of polar sheathed flagella. Their cell envelope has a characteristic Gram-negative structure, but many other components show unique features adapted to the habitat of *H. pylori* in the human stomach [6]. Compared to other pathogenic bacteria, *H. pylori* features a small genome (~1.6-Mbp) consisting of a single circular chromosome that encodes ~1600 proteins [7].

In 1983, *Helicobacter pylori* was successfully isolated for the first time from gastric mucosa biopsies of patients with chronic antral gastritis [8]. Subsequent studies demonstrated a strong association between *H. pylori* infection and chronic gastritis, with compelling evidence linking the bacterium to peptic and duodenal ulcer disease, as well as an increased risk of gastric cancer [9]. In recognition of this carcinogenic potential, the International Agency for Research on Cancer (IARC) classified *H. pylori* infection as a Group I carcinogen in 2014 [10].

*Helicobacter pylori* is a highly adapted gastric pathogen capable of colonizing the mucus layer of the human stomach. Its persistence within this niche is mediated by multiple virulence factors, including motility, urease activity, and adhesion mechanisms [6,11]. Urease is indispensable for colonization, as it hydrolyses urea into ammonia and carbon dioxide, thereby neutralizing the gastric acidity in the surroundings of the bacteria, and promoting survival of the bacteria during transient exposure to the hostile luminal environment. In addition, urea serves as a critical nitrogen source for bacterial metabolism [12]. Adhesion is another critical factor, as *H. pylori* use outer membrane proteins (adhesins) to attach to receptors on gastric epithelial cells. This attachment allows the bacteria to resist challenges such as epithelial turnover, mucus shedding, and the mechanical forces of gastric emptying [13]. In strains carrying the cag pathogenicity island (cagPAI), adherence is followed by activation of a type IV secretion system (T4SS), which injects effector molecules, most notably the cytotoxin-associated gene A (CagA) protein, into host cells [14]. Once phosphorylated, CagA interacts with multiple intracellular targets, disrupting signalling pathways and contributing to increased cell motility, loss of tight junction integrity, DNA damage, and even oncogenic transformation [15]. Many strains also produce a vacuolating cytotoxin A (VacA), which is a pore-forming autotransporter protein that modulates host immune responses and facilitates tolerance to infection. While VacA is not essential for colonization, its role in disease progression remains under debate [16]. Colonization invariably triggers a pro-inflammatory response, with gastric epithelial cells recruiting immune cells into the submucosa, thus leading to chronic gastritis. Although this inflammation is often asymptomatic and can persist for decades, its intensity varies depending on the bacterial strain, host genetics, and environmental factors [17]. Among virulence determinants, a functional cagPAI is considered the strongest driver of inflammation [18]. Over time, sustained gastritis may progress to gastric atrophy and even gastric adenocarcinoma [6].

Although gastritis develops in nearly all infected individuals, around 80% remain asymptomatic throughout life. The reasons behind this variability in clinical outcomes remain unclear [6]. Once acquired, *H. pylori* infection usually persists for the rest of the life unless treated by antimicrobial therapy [19]. Standard treatment for eradicating *H. pylori* is a triple therapy, which involves two antibiotics and a proton pump inhibitor (PPI) or ranitidine bismuth [20]. Common antibiotics include amoxicillin, tetracycline, metronidazole, or tinidazole, and macrolides such as clarithromycin or azithromycin [21]. However, rising antibiotic resistance has reduced eradication success rates in recent years [22]. Therefore, accurate diagnosis of infection and antibiotic susceptibility testing is a crucial measure before initiating therapy [23].

In clinical practice, *H. pylori* infection often remains undetected across all age groups. In children, naturally occurring acute infection is rarely identified, although it is presumed to manifest frequently as nonspecific abdominal symptoms of heterogeneous aetiology. Diagnostic approaches for *H. pylori* detection include both invasive and non-invasive methods, with the choice determined by the clinical context. Non-invasive techniques such as the 13C-urea breath test and stool antigen assays provide reliable detection of active infection [6]. Invasive diagnostics require endoscopic biopsy specimens, which enable histopathological assessment of gastritis severity and staging, as well as direct detection of *H. pylori* through polymerase chain reaction (PCR), microbial culture, rapid urease testing, or molecular analyses. Furthermore, antibiotic susceptibility testing can be performed from stool or biopsy samples using microbial culture, next-generation sequencing (NGS), or real-time PCR (RT-PCR), providing critical information for guiding eradication therapy [6].

*Helicobacter pylori* detection by culture is challenging due to the fastidious character of the bacterium that requires complex selective media containing numerous antibiotics and supplements, and microaerophilic conditions [24,25]. Furthermore, detection is hampered by the ability to readily enter a viable but non-culturable (VBNC) state, where the bacteria remain metabolically active but cannot be propagated in vitro [26,27]. It is thought that this ability represents an important strategy for bacterial survival in unsuitable conditions and to escape from the immune system [28]. Consequently, culture of *H. pylori* is characterized by a low sensitivity, with successful isolation strongly influenced by transport conditions, media quality, and laboratory expertise. Even in experienced laboratories, recovery rates range only between 50% and 70% of biopsy specimens from infected individuals [29]. Additional limitations of culture include its labour-intensive nature, high costs, and extended turnaround times. Nevertheless, culture remains the only method that permits phenotypic antimicrobial susceptibility testing, which is critical for guiding a tailored therapy [23]. In gastric biopsy specimens, the bacteria typically exhibit their characteristic spiral morphology with bluntly rounded ends. This morphology is also predominant in freshly established cultures. However, over time, the bacterium undergoes morphological transformation, first assuming a U-shaped rod form and eventually converting into coccoid cells. These coccoid forms are smaller, lack flagella, and represent the typical shape of the VBNC state of the bacteria [27]. Compared to spiral forms, the coccoid cells exhibit enhanced persistence under environmental stress, increased tolerance to antibiotics, and prolonged survival. Their poor resuscitation under laboratory conditions poses a major challenge to culture-based diagnostics and contributes to the variability in isolation success observed across clinical studies [26,27].

In a systematic review, Hooi et al. [30] quantified the global prevalence of *H. pylori* infection using regional estimates and reported that approximately 4.4 billion individuals were likely infected worldwide in 2015. This corresponds to nearly half of the global population. However, the worldwide prevalence demonstrates marked heterogeneity according to age, ethnicity, comorbid conditions, geographic region, socioeconomic status, and hygiene standards [6]. The majority of new infections are acquired during childhood, typically prior to the age of 10 years [31]. Furthermore, recent studies covering the period from 2014–2020 showed that prevalence in both paediatric and adult populations remains highest in low- and middle-income countries, particularly in Africa, the Eastern Mediterranean, Russia, and Latin America, whereas infection rates are lower in high-income countries and have declined in the Western Pacific region [32]. The prevalence of infection is higher in adults than in children. It is also higher in rural developing areas than in urban developed regions [33]. Prevalence of *H. pylori* infection in children has been decreasing owing to improvements in socioeconomic status and hygiene conditions. However, the global prevalence in children remained as high as 34% during 2014–2020 [32]. The higher prevalence in older individuals compared with children is explained by the fact that 90% of *H. pylori* infections are acquired during childhood and persist throughout life rather than by a higher risk of infection at an older age [6].

The transmission of *Helicobacter pylori* is proposed to occur through multiple pathways, with transmission from parent to child (person-to-person) being the most plausible and well-recognized route [34]. However, more recent investigations suggest that the acquisition of *H. pylori* may occur via multiple, more diverse pathways [35]. In particular, food and water are considered potential vehicles for the transmission of the pathogen [20,36,37], as many studies report the presence and survival of *H. pylori* or its DNA in food, water and feces from animals (Table 1, Table 2 and Table 3). Although *H. pylori* is unlikely to grow well, if at all, in most foods, the bacterium may survive for extended periods of time in low-acid/high-moisture environments under refrigerated storage [38].

This review provides a comprehensive synthesis of the current literature on the presence and survival of *H. pylori* in food and water, with a focus on evaluating the evidence for their potential role in *H. pylori* transmission. For this purpose, the available literature published from 1989 onwards was examined, together with relevant references included in respective publications. Furthermore, a manual review of the reference lists of the available primary and review articles was performed to identify relevant articles. Articles related to *H. pylori* transmission by water and food were identified in PubMed and Web of Science Core Collection. The retrieval deadline was August 2025. All articles retrieved by the database search were manually reviewed in order to decide on their relevance and suitability before they were included in the review. The following title keyword combinations have been used for searching the databases:
(pylori[Title]) AND water[Title]PubMed: 123Web of Science: 218(pylori[Title]) AND food[Title]PubMed: 58Web of Science: 75(pylori[Title]) AND foods[Title]PubMed: 12Web of Science: 97(pylori[Title]) AND milk[Title]PubMed: 56Web of Science: 75(pylori[Title]) AND dairy[Title]PubMed: 2Web of Science: 10(pylori[Title]) AND meat[Title]PubMed: 10Web of Science: 11(pylori[Title]) AND fish[Title]PubMed: 15Web of Science: 44(pylori[Title]) AND bovine[Title]PubMed: 26Web of Science: 34(pylori[Title]) AND beef[Title]PubMed: 4Web of Science: 5(pylori[Title]) AND cow[Title]PubMed: 4Web of Science: 11(pylori[Title]) AND cattle[Title]PubMed: 2Web of Science: 4(pylori[Title]) AND camel[Title]PubMed: 3Web of Science: 4(pylori[Title]) AND sheep[Title]PubMed: 8Web of Science: 14(pylori[Title]) AND goat[Title]PubMed: 2Web of Science: 6(pylori[Title]) AND pig[Title]PubMed: 18Web of Science: 80(pylori[Title]) AND poultry[Title]PubMed: 4Web of Science: 4(pylori[Title]) AND chicken[Title]PubMed: 3Web of Science: 4(pylori[Title]) AND vegetables[Title]PubMed: 5Web of Science: 15(pylori[Title]) AND lettuce[Title]PubMed: 2Web of Science: 3(pylori[Title]) AND fruits[Title]PubMed: 8Web of Science: 25(pylori[Title]) AND animals[Title]PubMed: 12Web of Science: 112(pylori[Title]) AND dogs[Title]PubMed: 7Web of Science: 53(pylori[Title]) AND cats[Title]PubMed: 13Web of Science: 45

## 2. Transmission Routes of *Helicobacter pylori* Infection

**Figure 1:**
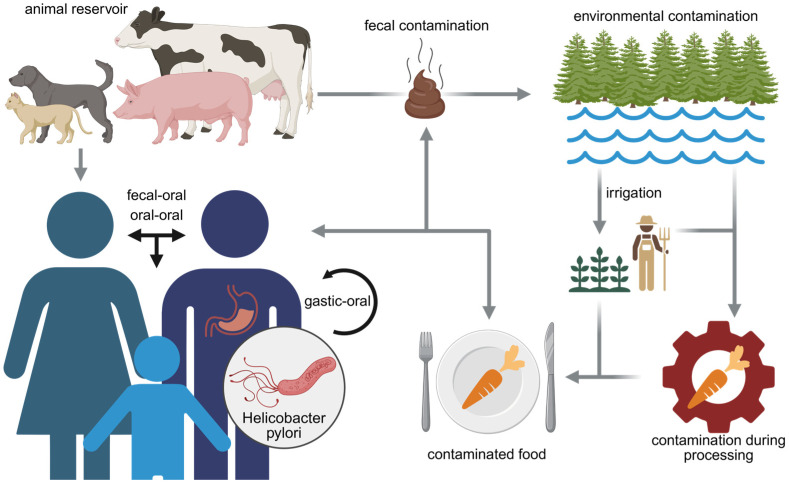
Potential transmission routes of *Helicobacter pylori* infection. Transmission routes with direct evidence are drawn in black arrows. Gray arrows indicate suspected transmission routes for which evidence is provided by the detection of *H. pylori* or its DNA in food and environmental samples. Oral-oral transmission is the most likely mode of person-to-person transmission, in particular within families, due to intimate contact between family members and the sharing of food utensils.

The pathways of *Helicobacter pylori* transmission still remain incompletely defined, and the relative contribution of different transmission routes has not been conclusively established [39]. Although multiple transmission routes for *H. pylori* infection have been proposed (Figure 1), person-to-person spread is still considered the most likely route of transmission [26,34]. Person-to-person transmission can be broadly distinguished into vertical transmission, denoting spread from parents to offspring within families, and horizontal transmission, referring to an acquisition of the pathogen through contact with non-family members or, potentially, via environmental contamination [40]. Evidence for vertical transmission includes the frequent detection of identical *H. pylori* strains among family members [41,42], with maternal-to-child transmission emerging as the predominant pathway within households [35,43]. Further support was derived from clinical studies showing clustering of infections among family members and the increased likelihood of infection in children of infected parents [44]. Genotyping analyses demonstrated strain concordance in 56% of mother–offspring pairs, but in none of the father–offspring pairs studied, and also in 81% of siblings [42]. Nevertheless, more recent investigations have challenged the primacy of parent–child transmission, suggesting instead that acquisition may occur via multiple, more diverse pathways [35].

Three possible routes of transmission from the stomach of one person to that of another have been described and are discussed below. Oral–oral, gastric–oral, and fecal–oral transmission is regarded as the most plausible person-to-person transmission routes [34]. Furthermore, sexual transmission of the pathogen has also been hypothesized [45]. The minimum infectious dose in humans remains uncertain; however, experimental challenge models for *H. pylori* infection in human volunteers suggested an infectious dose of about 105 colony-forming units (CFU) [46].

### 2.1. Oral–Oral Transmission

Oral–oral transmission is supported by the detection of *H. pylori* in saliva, dental plaque and subgingival biofilm by culture and PCR [47,48,49,50]. Importantly, *H. pylori* has been shown not only to colonize the stomach but also to persist in the oral cavity. Moreover, eradication therapy is generally more effective in the gastric niche than in the oral environment, suggesting that the oral cavity of patients who are gastric *H. pylori*-positive may serve as a potential reservoir, particularly in individuals with gingivitis or chronic periodontitis [51]. Evidence of shared or identical strains among couples further supports the possibility of direct person-to-person transmission or exposure to common environmental sources [42,52]. However, the frequent observation of unrelated strains between spouses provides a counterargument against oral–oral transmission as an exclusive route [33,53].

### 2.2. Gastric–Oral Transmission

Gastric–oral transmission of *H. pylori* is supported by successful culture of *H. pylori* from vomitus, which confirms that viable bacteria can be expelled during episodes of vomiting or gastric reflux [49]. Additional evidence comes from the isolation of *H. pylori* from gastric juice in symptomatic patients [54], as well as documented cases of transmission through gastric intubation [55]. Epidemiological studies further reinforce this route by showing an association between childhood infection and exposure to siblings or household members experiencing vomiting or gastroenteritis [56,57]. Collectively, these findings implicate vomitus as another significant vehicle for transmission, particularly under conditions of poor hygiene [33].

### 2.3. Fecal–Oral Transmission

Fecal–oral transmission of *H. pylori* is hypothesized to occur through ingestion of contaminated food, water, or other material under conditions of poor sanitation. This route is supported by the frequent detection of *H. pylori*-specific DNA in human fecal samples [58,59,60,61], along with results from animal model experiments demonstrating transmission in settings conducive to fecal–oral spread but not oral–oral spread [62]. In this animal model, *H. pylori-*inoculated mice were housed together with non-inoculated mice, either in cages without grating, which would facilitate fecal–oral transmission and oral–oral transmission, or in cages with grating, which would facilitate only oral–oral transmission. The exclusive isolation of *H. pylori* from the non-inoculated mice housed in the cage without grating supports the fecal–oral route over the oral–oral [62]. The reported occurrence of *H. pylori* infections due to transmission via fecal contaminants among institutionalized young people during outbreaks of gastroenteritis [63] further supports the fecal–oral route in the transmission of *H. pylori*. Hence, the fecal–oral route of *H. pylori* transmission seems to be particularly relevant when the hygienic conditions are poor.

Taken together, *H. pylori* can be cultivated from vomitus, stool and saliva of individuals with infection, which demonstrates the transmissibility of *H. pylori* via these routes [49]. Oral–oral and fecal–oral routes are still considered the most likely routes of transmission [34,64]. In particular, fecal–oral transmission of *H. pylori* has important implications because the bacteria may occur in food and water supplies subsequent to fecal contamination [65]. This opens the possibility of various environment-to-person transmission routes that also may include water or food as vehicles of transmission.

## 3. The Role of Water in the Transmission of *Helicobacter pylori*

### 3.1. Survival of Helicobacter Pylori in Water

Given its important implication for environmental transmission of *H. pylori* and public health, the potential for *H. pylori* to survive and persist in water has been the subject of extensive investigations (Table 1). Early evidence was provided already in 1990 by West et al. [66], who demonstrated the survival of *H. pylori* at 7 °C for 3–7 days in artificial seawater and for 11–14 days in distilled water. In physiological saline, *H. pylori* survived up to 16 days. A marked effect on bacterial survival was reported for the incubation temperature, as the bacteria lost their culturability within 1 day in distilled water and within 3 days in saline when incubated at room temperature [66]. These findings highlighted that cooler conditions may significantly prolong survival, and subsequent studies corroborated these observations. Fan et al. [67] confirmed survival at 4 °C for up to 4 days in tap water, albeit with a steady decline in colony-forming units and Nayak et al. [68] reported that *H. pylori* remained culturable for 120 h at 4 °C as opposed to only 24 h at 15 °C. A study by Konishi et al. [69] demonstrated that *H. pylori* strains stored in deep groundwater or seawater at 4 °C remained culturable for at least 7 days, and, notably, exhibited superior maintenance of their spiral morphology and culturability compared with controls in nutrient-rich *Brucella* broth. This unexpected result suggested that natural waters may provide more favourable conditions for *H. pylori* survival and persistence than artificial laboratory media (*p* < 0.01) [69]. However, the idea that low temperatures are essential was challenged by Azevedo et al. [39], who demonstrated that *H. pylori* survived for more than 96 h at 25 °C. The hypothesis that low temperatures may significantly prolong survival was also questioned by a study from Boehnke et al. [70], who reported a statistically significant relationship between lower temperatures and a lower likelihood of the presence of *H. pylori* in drinking water. Interestingly, among several *Helicobacter* species tested, *H*. *pylori* showed the greatest resilience under aquatic conditions [39].

A consistent observation across the survival studies has been the morphological transition of *H. pylori* cells from spiral rods to coccoid forms as culturability declines, reflecting entry into a VBNC state. In 2003, it was observed that, although cultivability decreased, morphological heterogeneity persisted, with coccoid cells predominating in laboratory-induced VBNC states, while environmental samples contained a mixture of the forms. Importantly, VBNC forms appeared to be metabolically active, raising concern that they may remain infectious despite being undetectable by standard culture techniques [71]. In support of this assumption, Moreno et al. [72] reported that *H. pylori* could survive chlorination in drinking water in the VBNC state, thereby escaping disinfection and potentially reaching consumers undetected by conventional microbiological assays. Shahamat et al. [73] used autoradiographic approaches to further confirm the persistence of *H. pylori* in water in a metabolically active but non-replicating condition, thus pointing to a potential role as a waterborne pathogen.

**Table 1:** Studies on the survival of *Helicobacter pylori* in food and water.

Year	Title	Result	Reference
1989	Survival of *Campylobacter* *pylori* in artificially contaminated milk	*H. pylori* did not multiply in milk but survived with a steady decline by one log in four days at both temperatures. At room temperature *H. pylori* was detectable after 5 days, and at 4 °C, over the whole test period of 6 days.	Karim et al. [74]
1990	Survival of *Helicobacter* *pylori* in water and saline	At 7 °C *H. pylori* remained viable and culturable for a period of 3–7 days in artificial seawater, 11–14 days in distilled water and up to 16 days in physiological saline. At room temperature *H. pylori* became non- culturable within 1 day in distilled water and 3 days in saline.	West et al. [66]
1993	Use of autoradiography to assess viability of *Helicobacter pylori* in water	*H. pylori* remained viable and culturable for up to 48 h and, in some cases, 20 to 30 days, depending on physical conditions of the environment.	Shahamat et al. [73]
1998	Survival of *Helicobacter pylori* in milk and tap water	*H. pylori* survived for up to 10 days in milk at 4 °C storage but only 4 days in tap water.	Fan et al. [67]
1998	Biofilms in drinking water systems: a possible reservoir for *Helicobacter pylori*	Artificially inoculated *Helicobacter pylori* were detected in biofilm material for a period of up to 192 h.	Mackay et al. [75]
2000	Survival of *Helicobacter* *pylori* in beef products	*H. pylori* survived in ground beef stored at 4 °C for 6 days in contact with air and for 3 days after vacuum packaging. In ground beef stored at −18 °C packaged either in air or in vacuum, *H. pylori* survived for 3 days.	Stevenson et al. [25]
2001	Survival of *Helicobacter* *pylori* in ready-to-eat foods at 4 °C	*H. pylori* survived in pasteurized milk and tofu up to 5 days but only 2 days in leaf lettuce and raw chicken. No survival was observed in yogurt.	Poms et al. [65]
2002	Optimizing enrichment culture conditions for detecting *Helicobacter pylori* in foods	*H. pylori* survived 6 days in sterile milk at 4 °C, 3 days in autoclaved ground beef, and 7 days in irradiated ground beef.	Jiang and Doyle [24]
2003	Survival of *Helicobacter* *pylori* in a natural fresh- water environment	*H. pylori* survived in VBNC state.	Adams et al. [71]
2004	Fate of *Helicobacter pylori* artificially inoculated in lettuce and carrot samples	*H. pylori* survived for up to 72 h in sanitized lettuce and carrot and up to 96 h in sterilized carrot samples.	Gomes et al. [76]
2007	Survival and viability of *Helicobacter pylori* after inoculation into chlorinated drinking water	*H. pylori* lost culturability after 5 min in chlorinated drinking water. Viable cells were still detected after 3 h but not after 24 h.	Moreno et al. [72]
2007	Survival of *Helicobacter* *pylori* in artificially contaminated ultrahigh temperature and pasteurized milk	*H. pylori* survived up to 9 days in pasteurized milk and 12 days in UHT milk.	Quaglia et al. [77]
2008	Survival of gastric and enterohepatic *Helicobacter* spp. in water: implications for transmission	*H. pylori* survived in water at 25 °C in the dark > 96 h, whereas *H. felis* survived < 6 h.	Azevedo et al. [39]
2008	Persistence of *Helicobacter* *pylori* in heterotrophic drinking-water biofilms	Culturable *H. pylori* could not be cultured at any time point but was able to persist as VBNC in biofilms for at least 31 days.	Giao et al. [78]
2010	Survival of spinach- associated *Helicobacter* *pylori* in the VBNC state	*H. pylori* introduced to spinach rapidly became non- detectable by plating, but mRNA transcripts were still detectable after 6 days.	Buck et al. [79]
2011	Survival of *Helicobacter* *pylori* in Turkish fermented sucuk and heat-treated sucuk during production	*H. pylori* could grow and survive during traditional sucuk fermentation and drying.	Guner et al. [80]
2017	Biofilm formation enhances *Helicobacter pylori* survivability in vegetables	*H. pylori* survived for up to 3 days in spring onion, lettuce and spinach and 4 days in cabbage.	Ng et al. [81]
2020	Survival of *Helicobacter* *pylori* as culturable and non-culturable form in artificially contaminated *Mytilus galloprovincialis*	*H. pylori* survived in artificially contaminated mussels. The bacteria could be isolated after 2 days, and after 4 days, detected as a non-culturable form.	Quaglia et al. [82]
2022	Isolation of *Helicobacter* *pylori* from raw milk and study on its survival in fermented milk products	*H. pylori* remained viable for two days in yoghurt without probiotics and survived for 7 days in control milk samples.	Al Sherief et al. [83]
2022	Exposure to water results in lysis and death of *Helicobacter pylori*	*H. pylori* rapidly lose their growth ability, lyse and die after exposure to sterile distilled water, making it unlikely that *H. pylori* survives in the VBNC state in water.	Inamasu et al. [84]

UHT: Ultra Heat Treated; VBNC: Viable But Non-Culturable.

Despite compelling evidence for survival in water under certain conditions, not all studies support the persistence of *H. pylori* in the VBNC state. Inamasu et al. [84] investigated the morphological and physiological changes after exposure of spiral *H. pylori* to sterile distilled water at 37 °C and observed that approx. 95% of spiral cells disappeared within one day, while coccoid forms increased. Monitoring the bacteria by fluorescence and electron microscopy revealed progressive cell deformation, collapse, and leakage. Viability assays indicated rapid loss of growth capacities, with continued decline in total bacterial counts over time. These observations suggest that, at least under some conditions, transformation into coccoid forms represents a degeneration process, rather than a survival strategy, and that water exposure may be lethal rather than conducive to the survival and persistence of *H. pylori*.

### 3.2. Association of Helicobacter Pylori with Water

Multiple studies provide evidence for the presence of *H. pylori* in water, either by isolation of *H*. *pylori* or detection of its DNA in various types of water sources, including drinking water, well water, surface water, wastewater, and sewage (Table 2). Direct monitoring of coccoid forms in environmental samples [85], combined with experimental demonstrations of bacterial persistence in artificially contaminated water [39,69,71,73], is supporting the hypothesis that inadequately treated water may facilitate transmission of the pathogen. This is biologically plausible, as *H*. *pylori* colonizes the human stomach and is shed in the stool of infected individuals [49], making wastewater and sewage logical reservoirs of the organism.

The majority of investigations into waterborne *H. pylori* have relied on molecular methods, particularly PCR-based assays. For example, Nayak et al. [68] detected *H. pylori*-specific DNA in 86% (20/23) of sewage samples by quantitative polymerase chain reaction targeting a specific fragment of the *vacA* gene. In contrast, Yáñez et al. [86] reported a lower detection rate, with only 3 of 40 wastewater samples positive by real-time PCR amplifying a 102-bp fragment of the *cagE* gene. Another study reported detection of *H. pylori* in diverse aquatic environments and sewage sludge. Amplification of the 16S rRNA gene was achieved in 14 of 39 wastewater samples, whereas PCR amplification of the *ureA* gene yielded only two positive samples [87]. Although studies using a PCR-based detection approach predominate, few studies also reported the successful cultivation of *H. pylori* from wastewater samples. A study by Lu et al. [88] demonstrated the presence of viable *H. pylori* in sewage by isolating and genotyping 23 cultures of *H. pylori* from untreated municipal wastewater. However, another investigation that applied a multimodal detection strategy, combining PCR, culture, and fluorescence *in situ* hybridization (FISH) for the analysis of water and wastewater samples, failed in isolating viable *H. pylori*. While FISH identified *H. pylori* in two river water and one wastewater sample, and PCR detected the pathogen in one sample, culture was negative for all samples [89]. Collectively, these findings provide converging evidence that *H. pylori* not only transiently contaminate wastewater and sewage but may also persist in these environments. The pathogen appears to survive both in a VBNC state and, under some circumstances, as active, culturable bacteria. These observations point to a role of wastewater and sewage as a potential reservoir and source for *H. pylori*.

Due to the strong association of *H. pylori* with the human gastrointestinal tract and its fecal shedding, it is not surprising that the bacterium is often detected in wastewater and sewage. However, its occurrence is not restricted to these aquatic environments, as the bacterium, or at least its DNA, has been detected in diverse aquatic habitats, including surface waters such as rivers, lakes, and seawater [58,90,91,92,93,94,95,96].

In coastal marine environments, *H. pylori* DNA was detected in 41.7% of seawater and plankton samples collected from the Straits of Messina in Italy, although no culturable bacteria were recovered [90]. Similarly, nested PCR analyses of seawater along the Adriatic coast of Italy revealed the presence of both free-living and plankton-associated *H. pylori* DNA [91]. Subsequent studies went further and were even able to isolate *H. pylori* from marine zooplankton [92]. Interestingly, these isolates, along with the reference strain ATCC 43629, could be reactivated from the VBNC state only, when incubated together with the marine copepod *Tigriopus fulvus*, to whose surface the bacteria adhered. This finding suggests that copepods may play a vital role in the persistence and potential transmission of *H. pylori* in seawater [92]. Widespread occurrence of *H. pylori* in marine and estuarine systems has also been reported for North America and the Caribbean, as *H. pylori* could be detected in freshwater, estuarine, and beach sites in Delaware [96], and from coastal and riverine sites in Georgia, Trinidad, and Puerto Rico, including Maracas River and Ceiba Creek [94]. These observations suggest a broad geographic distribution of *H. pylori* in subtropical and tropical coastal waters. Notably, no significant correlation was observed between *H. pylori* and conventional fecal indicator organisms such as *E. coli* and enterococci in these studies, which indicates an absence of fecal pollution in the sampled water. Detection in freshwater sources has been equally notable. In 1999, a study identified actively respiring *H. pylori* binding monoclonal anti-*H. pylori* antibody in 60% of surface water samples and 65% of shallow groundwater samples [93]. Furthermore, direct viable count fluorescence *in situ* hybridization (DVC-FISH) identified viable *H. pylori* cells in 47.9% of samples from the Aliakmon River, which is an important potable water source in Greece. No seasonal variation was observed, nor was there a correlation with conventional indicators of fecal contamination [95]. Conversely, in Japanese rivers, *H. pylori* DNA was detected only in middle and downstream sections, corresponding to areas with human settlement and suggesting that anthropogenic activity may influence the distribution of the pathogen [58]. Another study from Japan conducted an investigation in a high-prevalence region that analyzed environmental samples, including tap and well water, field soil, river and pond water, flies, and bovine feces, which were collected from areas surrounding the residences of individuals enrolled in an epidemiological survey. Nested PCR assays detected *H. pylori*-specific DNA in water, soil, flies, and bovine feces, supporting the hypothesis that *H. pylori* is ubiquitously present in the natural environment and that contaminated water may serve as a transmission route [97]. More recent analyses using viability qPCR in northeast Spain demonstrated a particularly high prevalence, with viable *H. pylori* detected in 91.3% of surface water samples at mean concentrations of 3.46 ± 1.06 log cells/100 mL [98]. These data indicate that urban surface waters may contain substantial populations of viable *H. pylori* and represent both a potential reservoir for transmission and a public health concern. Further evidence was provided by the widespread detection of *H. pylori* DNA in environmental samples worldwide [99], and experimental studies that demonstrated survival and persistence of *H. pylori* in water for prolonged periods, often in the VBNC state.

Epidemiological studies have indicated non-hygienic drinking water and inadequate sewage disposal as key factors contributing to the contamination or recontamination of water supplies and a subsequent infection risk [33]. First evidence for the presence of *H. pylori* in drinking water was shortly reported after its identification as the causative agent of peptic ulcer disease and a risk factor for gastric cancer. In 1996, Hultén et al. [100] analyzed 48 drinking water samples from locations near Lima, Peru, detecting the *H. pylori* adhesin gene in 24 samples by PCR, while 16S ribosomal RNA, as a marker for viable bacteria, was amplified by reverse transcription PCR (RT-PCR) in 11 of the 48 samples. These findings confirmed the presence of *H. pylori* in Peruvian drinking water and supported previous epidemiological data from the same population, reinforcing the concept of waterborne transmission in endemic settings. Fujimura et al. [101] further demonstrated the epidemiological relevance of water sources by comparing three Japanese populations exposed to different drinking water supplies: two using river water and one using groundwater. The population consuming groundwater exhibited a significantly lower prevalence of *H. pylori* infection. The importance of water treatment was also highlighted in an analysis of five water systems in the Mexico City area. Prior to treatment, the prevalence of *H. pylori* 16S rRNA gene detection in dam water samples was 100%, while post-treatment samples revealed zero prevalence, clearly indicating the effectiveness of water purification processes in eliminating *H. pylori* [102]. However, in contrast to these findings, a study from Dhaka, Bangladesh, reported absence of *H. pylori* DNA in drinking water samples, despite the confirmed presence of other pathogenic bacteria detected by the same highly sensitive real-time PCR assays [103]. Although this result suggests that properly treated drinking water may not represent a significant transmission pathway in all geographic contexts, several other studies demonstrated that *H. pylori* may survive conventional drinking water disinfection practices, at least as a VBNC form. Moreno et al. [72] demonstrated that *H. pylori* persisted throughout standard water treatment processes and reached final distribution points in a VBNC state that was undetectable by culture-based techniques. Importantly, some other studies reported the detection of culturable, and thus viable, *H. pylori* in treated water. For example, in Bogotá, Colombia, *H. pylori* was identified by culture, quantitative PCR (qPCR), and fluorescence *in situ* hybridization (FISH) in 56 of 310 water samples collected from three drinking water treatment plants. Positive samples included not only influent water but also effluent and post-treatment water, providing direct evidence that potable water supplies can harbour viable *H. pylori* and may thus serve as a transmission route [85].

Several studies across diverse geographic regions further substantiated the detection of *H. pylori* DNA in untreated well water [104,105,106,107]. In the United States, molecular analyses revealed *H. pylori* in well water samples, with a correlation observed between the presence of the bacterium in wells, *H. pylori* infections among consumers, and detection of *Escherichia coli* as a fecal indicator [104]. In urban Japan, the *H. pylori* 16S rRNA gene was amplified from 2 of 6 water samples, including one from a well that was used by individuals with a history of *H. pylori* infection [105]. Another Japanese study employing magnetic-bead purification and PCR amplification detected the *ureA* gene in 4 of 43 well water samples (9.3%) and the 16S rRNA gene in one sample (2.3%) [106]. Similarly, a German investigation using PCR to analyse 157 private well samples from rural counties near Leipzig identified *H. pylori*-specific DNA in 10.8% of wells in one county and 9.2% in another, with an estimated bacterial density averaging 931 cells/L [107]. Collectively, these findings demonstrate that consumption of untreated well water may represent a significant risk factor for *H. pylori* infection, and that both VBNC and culturable forms of the bacterium may persist in drinking water systems, despite standard treatment interventions.

Most studies reporting the presence of viable *Helicobacter pylori* in drinking water intended for human consumption have been conducted in developing countries. A high prevalence was reported for Lahore, Pakistan, where the investigation of drinking water and clinical samples revealed presumptive *H. pylori* in 37.5% (225/600) of drinking water samples, with 40% (90/225) of these testing PCR-positive for the *vacA* and *cagA* genes [108]. In Basrah governorate, Egypt, analysis of 198 drinking water samples from 22 districts identified 14 isolates as *Helicobacter* spp., of which 10 were confirmed as *H. pylori*. A subsequent study in the same region reported *H. pylori* in 4.1% of 266 tap water samples and 1.5% of 205 reverse osmosis water samples [109,110]. In Iran, prevalence in municipal and dental water systems ranged from 2.5% to 5.8% across Isfahan [111], while broader surveys of four provinces confirmed viable *H. pylori* in 3% of drinking water samples, with genotyping revealing identical strains in water and human clinical isolates [112]. Additional studies isolated the bacteria from 4% of tap water samples from Kermanshah [113] and even in bottled mineral water (1.77%), with isolated strains displaying virulence and antimicrobial resistance [114]. In Yemen, 9.6% of tap water samples tested positive by culture, with 85.7% of detections occurring in late winter and spring [115]. Viable *H. pylori* has also been reported in South America and in Europe, in 6 of 24 samples from a public drinking water system in eastern Spain using propidium monoazide quantitative polymerase chain reaction (PMA-qPCR) and direct viability count fluorescence *in situ* hybridization (DVC-FISH) [116].

However, most studies rely solely on molecular detection of *H. pylori* DNA, without confirming viability. In Canada, DNA was detected in water delivery truck samples [117]; in Karachi, Pakistan, only 4% of the analysed drinking water samples were positive by PCR [118]; and in Egypt, 2 of 50 samples (3.92%) tested positive in the *ureC* gene PCR [119]. In Peru, PCR detection in tap water revealed higher percentages with 12.2% positive samples from cancer patient households [120] and up to 20.3% positive tap water samples from Lima analysed by qPCR [70]. In Kermanshah, Iran, *H. pylori* DNA was detected in 56% of samples, with higher prevalence in well water (41/48) than in tap water (25/70) [121].

Biofilm formation appears to be an additional factor supporting the environmental survival of *H. pylori*. Biofilms, which are ubiquitous in natural aquatic ecosystems, provide a protective micro-environment that may enhance bacterial persistence. Analysis of water and biofilm samples from drinking water distribution systems in England detected *H. pylori* DNA in biofilm samples, although viable bacteria were not recovered [122]. *Helicobacter* species-specific DNA was identified in 26% of 151 samples from domestic properties, schools, and hydrants, with the highest frequency observed in biofilms (42%). Culture-based studies have also reported the presence of *H. pylori* in biofilms, but with a much lower prevalence. In the Junin region of Peru, *H. pylori* was isolated from 2 of 192 (1.04%) tap water samples and 3 of 192 (1.56%) tap biofilm samples [123]. Laboratory investigations further demonstrated that *H. pylori* can persist in biofilms for extended periods, ranging from days to weeks [75,78]. Using a laboratory model with removable stainless-steel coupons, *H. pylori* NCTC 11637 was detected by PCR in biofilm material for up to 192 h, far exceeding theoretical washout times and suggesting that biofilms can serve as stable reservoirs [75]. A study by Giao et al. [78] extended these findings by showing that biofilms may not only shelter *H. pylori* but also act as concentration points. Upon sloughing, biofilm fragments released viable bacteria, potentially bypassing routine microbiological surveillance and posing a public health risk. Additionally, biofilm formation has been shown to enhance the survival of *H. pylori* on vegetables [81]. Collectively, these findings indicate that biofilms on surfaces within water distribution systems may act as sites for passive accumulation or as reservoirs capable of sustaining potentially infectious *H. pylori*.

Another potential environmental niche for *H. pylori* is free-living amoebae (FLA). These protozoa are widespread in natural and artificial environments, typically grazing on bacteria. However, some bacterial pathogens can resist predation by amoeba, surviving intracellularly or even proliferating within the host [124]. Early work by Winiecka-Krusnell et al. [125] demonstrated successful co-cultivation of *H. pylori* with *Acanthamoeba castellanii* and more recently, Moreno et al. [126] showed that *H. pylori* can be internalized and remain viable within FLA isolated from vegetables. Such interactions may enhance bacterial survival and suggest that amoebae could serve as environmental reservoirs and vehicles for transmission. Nevertheless, the role of amoebae as environmental hosts for *H. pylori* remains controversial, as a study examining the co-occurrence of *Helicobacter* spp. and *Acanthamoeba* in river and soil environments detected *Helicobacter* in river water but not in soil. This lack of spatial overlap did not support a consistent environmental association between *Helicobacter* spp. and *Acanthamoeba* [127].

Overall, the current literature presents strong evidence for the presence of *H. pylori* in diverse water systems worldwide, with prevalence and detection methods varying significantly by region, water source, and season.

**Table 2:** Occurrence of *Helicobacter pylori* in water and feces.

Year	Sample type	Country	Method	Result	Reference
1996	Peruvian children 2 months to 12 years old	Peru	13C-urea breath test	Overall prevalence was 48%. Children from homes with external water sources three times more likely to be infected, compared to homes with internal water sources.	Klein et al. [128]
1996	Drinking water	Peru	PCR, RT-PCR for 16S rRNA	*H. pylori* adhesin gene amplified by PCR from 24 of 48 drinking water samples, whereas RT-PCR for *H. pylori* 16S rRNA and *H. pylori* adhesin gene PCR was positive in 11out of the 48 samples.	Hulten et al. [100]
1999	Surface water and shallow ground water	USA	fluorescent antibody (CTC) staining	Actively respiring *H. pylori* binding monoclonal antibody present in 25 of 42 (60%) of the surface water samples and 13 of 20 (65%) of shallow groundwaters samples.	Hegarty et al. [93]
1999	Water from a delivery truck and two lakes	Canada	Nested PCR	*H. pylori-*specific DNA detected in water from a delivery truck and two lakes.	McKeown et al. [117]
1999	Tap and well water, field soil samples, flies, feces from cows	Japan	Nested PCR	*H. pylori-*specific DNA detected in water, field soil, flies and cow feces.	Sasaki et al. [97]
2001	Well water	USA	PCR	*H. pylori*-specific DNA detected using molecular methods in untreated well water.	Baker et al. [104]
2001	Tap water, well water, river water, and sea water	Japan	PCR, qPCR for 16S rRNA	None of the samples of tap (10 samples), river (10 samples), or sea water (10 samples) positive for adhesin, *ureA* or 16S rRNA gene PCR. None of the 6 samples of well water positive for adhesin or *ureA* PCR, but 2 of 6 samples revealed a positive 16S rRNA PCR.	Horiuchi et al. [105]
2001	Water systems for human use	Mexico	PCR	*H. pylori*-specific nested PCR positive in 58 (42%) of 139 analyzed water samples.	Mazari-Hiriart et al. [102]
2002	Municipal wastewater	U.S.-Mexico border	Culture, PCR	132 isolates obtained by culture from municipal wastewater samples and 23 isolates could be identified as *H. pylori.*	Lu et al. [88]
2003	Well water	Japan	PCR	*H. pylori*-*ureA* gene detected in 4 out of 43 (9.3%) well water samples and *H*. *pylori* 16S rRNA gene detected in 1 out of 43 (2.3%) samples.	Imanishi et al. [106]
2003	River water and wastewater	Spain	FISH, PCR	*H. pylori* detected by FISH in two river water samples and one wastewater sample, while PCR yielded only one positive result. *H. pylori* culture negative for all samples.	Moreno et al. [89]
2004	Seawater	Italy	Nested PCR	*H. pylori,* either free or bound to planktonic organisms, detected in 7 out of 12 samples.	Cellini et al. [91]
2004	River water and stool samples from children living near the rivers	Japan	Nested PCR	*H. pylori* DNA detected in water from middle and downstream reaches of four rivers. Prevalence of *H. pylori* in stool samples from 224 children examined was 9.8% for those living near the middle reaches and 23.8% nearby downstream.	Fujimura et al. [58]
2004	Water from private wells	Germany	PCR; 13C-urea breath test	*H. pylori* DNA detected in about 10% of 157 wells. About 6% of 1884 tested children positive for *H*. *pylori*.	Krumbiegel et al. [107]
2004	Drinking water and biofilm samples	England	Culture, PCR	Culture negative for all 151 samples, but *Helicobacter*-specific DNA detected in 26% of samples from domestic properties, schools and hydrants, with the highest frequency in biofilms (42%).	Watson et al. [122]
2005	Seawater and plankton	Italy	Culture, PCR	*H. pylori* not detected by culture in any of 36 environmental samples, while 15 out of 36 (41.7%) samples were positive for the 16S rRNA gene. Amplification of the *ureA* gene was positive in 22.2% environmental samples and *cagA* PCR resulted in 19.4% positive samples.	Carbone et al. [90]
2005	Seawater	Italy	Culture, PCR	*H. pylori* isolated from water samples containing large zooplanktonic organisms.	Cellini et al. [92]
2005	Human stool and water	Spain	Nested PCR, stool antigen test (HpSA)	*H. pylori* detected in 33% of 36 human stool samples and in 66% of 15 wastewater samples, and in 11% of 23 river samples, but in none of the 19 spring water samples.	Queralt et al. [61]
2007	Wastewater	USA	Culture, PCR, qPCR	*H. pylori* DNA detected by qPCR in 86% (20/23) of sewage samples. In seeded groundwater *H. pylori* was detectable for up to 12 days by conventional MPN-PCR.	Nayak et al. [68]
2009	Water and biofilm samples	Bangladesh	Real-time PCR	*H. pylori* DNA not detected by real-time PCR in samples of drinking and environmental water (n = 75) and natural water biofilms (n = 21).	Janzon et al. [103]
2009	Potable water, surface water, and wastewater	Spain	Real-time PCR	*H. pylori* DNA detected in 3 out of 40 wastewater samples. All river (19) and drinking water (10) samples negative.	Yáñez et al. [86]
2010	Drinking water	Iraq	Culture	469 isolates obtained from 198 drinking water samples. Of 173 isolates tested, 14 isolates represented *Helicobacter* spp. and 10 identified as *H. pylori*.	Al-Sulami et al. [110]
2011	Drinking water	Pakistan	Culture, PCR	Presumptive *H. pylori* isolates obtained from 37.5% (225/600) of drinking water samples. 40% (90/225) of positive samples were PCR-positive for *vacA* and *cagA.*	Samra et al. [108]
2011	Coastal freshwater, estuary, and marine waters	USA	Clone library analysis, PCR	*H. pylori* 16S rRNA gene amplified in approx. 21% of the samples. 80% of those samples also positive for *H. pylori* 16S rRNA gene.	Twing et al. [96]
2012	Drinking water	Iraq	Culture	*H. pylori* isolated from 11 (4.13%) out of 266 tap water samples and 3 (1.46%) out of 205 reverse osmosis water samples.	Al-Sulami et al. [109]
2012	Drinking water	Pakistan	PCR	*H. pylori* DNA detected in 2 out of 50 (4%) water samples	Khan et al. [118]
2013	Tap water, dental units’ water, and bottled mineral water	Iran	Culture, PCR	*H. pylori* isolated from 2 out of 50 tap water samples (4%), 2 out of 35 dental units’ water (5.8%) samples, and from 1 out of 40 (2.5%) water cooler samples. *H. pylori ureC* gene detected in 7% of water samples including tap water (10%), dental unit water (11.4%), refrigerated water with filtration, and 10% of water cooler samples.	Bahrami et al. [111]
2013	Seawater	Georgia, Puerto Rico, Trinidad	Culture, PCR	*H. pylori* detected in 4 out of 31 samples.	Holman et al. [94]
2014	Drinking water	Iran	PCR	*H. pylori* DNA detected in 56% (66/118) of water samples. Frequency of 36% (25/70) for tap water and 85% (41/48) for wells.	Amirhooshang et al. [121]
2014	River water	Greece	DVC-FISH	*H. pylori* detected in 23 out of 48 water samples (47.9%), while no seasonal variation and no correlation with indicators of fecal contamination were observed.	Tirodimos et al. [95]
2015	Ground water, river water, tap water, and human blood	Egypt	PCR, ELISA	*H. pylori* DNA detected in 2 out of 51 (4%) water samples. ELISA test positive in 67% of 173 blood samples.	El-Sharouny et al. [119]
2015	Drinking water	Spain	Culture, qPCR, DVC-FISH	*H. pylori* detected in 16 out of 24 drinking water samples. Viable cells detected in 6 samples.	Santiago et al. [116]
2016	Drinking water	Iran	Culture, PCR	*H. pylori* isolated from 12 out of 400 (3%) drinking water samples.	Ranjbar et al. [112]
2016	Bottled mineral water	Iran	Culture, PCR	*H. pylori* isolated from 8 (1.77%) out of 450 bottled mineral water samples.	Ranjbar et al. [114]
2018	Surface water	Spain	PMA-qPCR	Viable *H. pylori* detected in 91.3% of samples, with an average concentration of 3.46 +/− 1.06 log cells per 100 mL.	Acosta et al. [98]
2018	Drinking water samples	Peru	qPCR	*H. pylori* detected in 49 of 241 (20.3%) drinking water samples by qPCR.	Boehnke et al. [70]
2018	Influent and effluent water from drinking water treatment plants (DWTP)	Colombia	Culture, qPCR, FISH	*H. pylori* isolated from 56 of 310 influent and effluent water samples, in 26 out of 155 (16.8%) influent samples, and in 30 out of 155 (19.4%) effluent water samples. *H. pylori* DNA detected in 77 out of the 310 influent and effluent water samples.	Vesga et al. [70]
2019	Tap water and gastric tissue from cancer patients	Peru	*hspA* and *ureA* gene qPCR	*H. pylori* detected by qPCR in 69.5% of 82 gastric tissue samples and in 12.2% of 82 tap-water samples collected from the homes of cancer patients.	Castillo et al. [120]
2019	Drinking water, wastewater, and sewage sludge	Iran	Nested PCR for 16S rRNA gene, semi-nested *ureA* PCR	*H. pylori* 16S rRNA gene detected in 36% (14/39) of wastewater samples and 8% (2/25) of drinking water samples, while PCR detection of the *ureA* gene yielded only two positive results. None of the anaerobically digested sewage sludge samples positive for *H. pylori*.	Farhadkhani et al. [87]
2021	Human stool and gastric biopsies, feces from cow, buffalo, sheep, and camel, feces and saliva from dogs and cats	Egypt	16S rRNA gene PCR	*Helicobacter* spp. DNA detected in 13 of 29 (44.8%) of the human samples. *H. pylori* in 2 (15.4%), and *H. bovis* in 4 (30.8%) samples, 7 (53.9%) unidentified. In fecal samples from livestock animals *Helicobacter* spp. DNA was detected in 6 out of 15 (40% cattle), 4 out of 12 (33.3% buffalo), 2 out of 8 (25% sheep), and 2 out of 9 (22.2% camel). *H. pylori* not detected in samples from livestock animals. In pets, *Helicobacter* spp. DNA detected in 13 (37.1%) out of 35 samples from dogs and 5 (21.7%) out of 23 samples from cats. *H. pylori* not detected in pet samples.	Youssef et al. [129]
2023	Tap water and surface water	Yemen	Culture	*H. pylori* detected in 9.6% tap water samples and 13.2% surface water samples.	Almashhadany et al. [115]
2023	Tap water	Iran	Culture; qPCR	*H. pylori* detected in 3 out of 50 tap water samples before enrichment, and 6 positive by RT qPCR after enrichment. Two samples of *H. pylori* culture positive.	Hasanvand et al. [113]
2025	Tap water and tap biofilm	Peru	Culture	*H. pylori* isolated from 2/192 (1%) tap water and 3/192 (1.6%) biofilm samples.	Custodio et al. [123]

16S rRNA: 16S ribosomal Ribonucleic Acid. ELISA: enzyme-linked immunosorbent assay; CTC: cyanoditoyl tetrazolium chloride; DVC-FISH: direct viable count fluorescence *in situ* hybridization; DWTP: drinking water treatment plants; FISH: fluorescence *in situ* hybridization PCR: polymerase chain reaction; MPN-PCR: most probable number polymerase chain reaction; PMA-qPCR: propidium monoazide quantitative polymerase chain reaction; qPCR: quantitative polymerase chain reaction; RT-PCR: reverse transcription polymerase chain reaction.

## 4. The Role of Food in the Transmission of *Helicobacter pylori*

### 4.1. Milk and Dairy Products

The potential role of food as a transmission vehicle for *H. pylori* has been the focus of extensive investigation over the past several decades (Table 3). Early indications of *H. pylori* presence in food originated from studies on raw milk. In 1999, Dore et al. [130] reported detection of the *H. pylori* 16S rRNA gene in 60% of 51 raw sheep milk samples using PCR. The *vacA* gene was amplified from five samples, while viable *H. pylori* was successfully isolated from only one sample. A subsequent study by the same group revealed detection of the 16S rRNA gene in 60% (38/63) of milk samples and 30% (6/20) of gastric tissue samples from sheep, with the *vacA* gene amplified only in five milk and two gastric tissue samples. Thus, suggesting that 16S rRNA gene amplification lacks specificity for *H. pylori* [131]. In Japan, Fujimura et al. [132] examined raw and pasteurized cow milk using semi-nested PCR, culture, and electron microscopy. The *ureA* gene was detected in 72.2% of raw milk and 55% of commercial pasteurized milk samples via semi-nested PCR. Immunomagnetic separation with *H. pylori*-specific antibodies followed by electron microscopy confirmed the presence of bacterial structures exhibiting specific immunoreactivity. Nevertheless, viable *H. pylori* was isolated from only one raw milk sample, with none recovered from pasteurized milk, implying that PCR detection may reflect free bacterial DNA or non-viable cells in processed dairy products [132]. Similarly, a study conducted in southern Italy identified the *glmM* gene of *H. pylori* in 34.7% (139/400) of raw milk samples from cows, goats, and sheep, marking the first report of *H*. *pylori* DNA in raw goat milk [133]. However, as in previous studies, no viable isolates were recovered. The failure to isolate viable *H. pylori* from milk by culture is reported by several studies. Despite optimized enrichment culture methods, the pathogen could not be recovered from 120 raw bovine milk samples [24] or 440 raw sheep milk samples from Turkey [134]. Similarly, bulk tank milk from northern Italy and raw cow and goat milk from the Czech Republic contained PCR-positive DNA but yielded no viable isolates [135,136]. These findings render the presence of viable *H. pylori* in milk questionable, although survival studies indicated that *H. pylori* can survive and persist in milk for 6–12 days at 4 °C [24,67,77]. However, the duration of survival in dairy products appears to depend on the product type. While *H. pylori* could not be recovered from inoculated yogurt [65], fermented milk products containing probiotics supported survival for up to 2 days [83].

Frequent occurrence of *H. pylori* in milk and dairy products is reported by several large-scale studies from developing countries. A large study from Iran reported PCR detection of the *ureC* gene of *H. pylori* in 12.5% (56/447) of bulk milk samples from bovine, buffalo, camel, ovine, and caprine herds, with culture-positive isolates obtained from only 3 samples (0.67%) [137]. Another study detected *H. pylori* in 19.8% of 520 raw milk samples and 19.2% of 400 traditional dairy products, with ovine milk (35%) and traditional cheese (30%) most frequently contaminated [138]. Additional Iranian studies reported between 13 and 16% prevalence for *H. pylori* DNA in raw bovine milk and dairy products, with culture confirming their viability in a minority of samples [139,140,141,142,143,144]. Detection of *H. pylori* antigen in milk and feces further supported milk as a potential source of exposure [145]. A study from Khartoum State, Sudan, detected the *H. pylori-glmM* gene in 22% of raw milk samples by nested PCR [146]. In Egypt, the detection of *H. pylori* by PCR ranged from 11% to 51.4% depending on the study and animal species, with higher prevalence in cows than in buffaloes or sheep. The results further suggested that some cows shed *H. pylori* in feces, which may also enter the milk [147,148]. More recent studies reported the presence of viable *H. pylori* in raw cow milk (13.3%) and marketable milk (6.6%), although PCR confirmed only 50% of presumptive isolates as *H. pylori* [83]. Another study reported detection of the *glmM* gene by PCR in 13% (5/13) of raw milk samples from animals showing a positive *H. pylori* stool antigen test [149]. Molecular and serological studies from Algeria detected the *glmM* gene in 13% of cow milk and a correlation with IgG positivity [150]. In Greece, fluorescence *in situ* hybridization detected *H. pylori* in 20% of raw bovine milk samples [151].

As pasteurization effectively inactivates *H. pylori* in milk, only unpasteurized or raw milk could represent a potential vehicle for transmission. The available data suggest that viable *H. pylori* occur primarily in raw, unpasteurized milk and that the potential for survival is strongly influenced by temperature, storage conditions, and microbial competition. Hence, raw milk could not be ruled out as a possible minor and context-dependent transmission route for *H*. *pylori*, particularly in settings with poor hygiene and/or widespread consumption of unpasteurized dairy products. However, while the cumulative evidence supports the plausibility of milk-borne exposure, most findings are derived from PCR-based studies with limited culture confirmation and scarce epidemiological corroboration. These studies demonstrate that *H. pylori* genetic material is frequently detectable in raw milk and dairy products, yet the recovery of viable organisms remains exceedingly rare. This discrepancy may be attributed to the bacterium’s transition into a VBNC state under environmental stress. Furthermore, the high sensitivity of molecular methods such as PCR may result in the detection of DNA from non-viable bacterial cells degraded during processing, free *H. pylori* DNA contained by the samples, or the presence of cross-reactive DNA sequences in complex food matrices, which further complicates interpretation of the data. Consequently, while these findings indicate at least a potential occurrence of *H. pylori*, definitive evidence for *H*. *pylori* transmission via milk and dairy products remains inconclusive and warrants further methodological refinement for reliable isolation and viability assessment.

### 4.2. Meat

Indirect evidence for *H. pylori* transmission via meat and meat products is provided by multiple studies demonstrating the presence of *H. pylori* in various types of meat [142,144,152,153,154,155,156,157,158,159,160,161,162] and the potential survival of the pathogen in meat under different storage and processing conditions [25,80].

Survival of *H. pylori* in raw meat was first demonstrated by a study from the United States examining rumen and abomasum mucosal samples from 105 cattle and 20 retail beef cuts. While isolation of the pathogen from the samples failed, spiking experiments demonstrated survival of *H. pylori* in ground beef samples [25]. At 4 °C, *H. pylori* survived for 6 days in contact with air and 3 days under vacuum packaging. At –18 °C, the bacteria survived for 3 days regardless of packaging conditions. Likewise, raw chicken meat supported the survival of *H. pylori* for up to 2 days at 4 °C [65]. Processed meat also revealed the property to promote survival of *H. pylori*, as the pathogen persisted for up to 7 days in Turkish fermented sausage (sucuk). The investigation revealed even growth of the bacteria during the fermentation process [80].

The presence of *H. pylori* DNA in meat samples has been reported by several studies. Meng et al. (2008) detected *H. pylori* DNA by multiplex PCR in 36% (4/11) of raw chicken samples and 44% (8/18) of ready-to-eat raw tuna meat from the Chicago area [162]. More recent studies from Egypt revealed a lower prevalence of *H. pylori* DNA. A study on 300 samples of chicken breast, liver, and gizzard samples, collected from retail shops in Qalyubia Governorate, detected *H. pylori* DNA in 5.3% of samples. In this study, two isolates were recovered from swabs of cutting boards [155]. Another study from Egypt also reported isolation of *H. pylori* from one sample each of raw meat, raw poultry meat, and luncheon meat [154], while *H. pylori* DNA was detected in 7.8% (7/90) of chicken meat, gizzard, and liver samples [159].

Isolation of viable *H. pylori* from meat is mainly reported in studies from developing countries. In raw chicken meat samples from Yemen and Iran, isolation of *H. pylori* was possible from 13.8% of 260 samples from Yemen and 6.3% of 320 samples from Iran, while no isolates were recovered from raw meat of goose or quail [152,153]. Further investigations in Iran revealed *H. pylori* in 26.2% (105/400) of raw meat from cow, sheep, goat, camel, and buffalo [142], and 8.7% (52/600) of raw meat samples, with ovine meat showing the highest prevalence (13%) [161]. A study comparing meat from butcheries and slaughterhouses in Iran reported 5% (11/220) overall, but with a markedly higher rate of contamination in slaughterhouse samples (72.2%) compared to butcheries (27.7%) [158]. A similar prevalence was found for hamburger and minced meat samples from Iran, with 7.3% (11/150) positive samples [157]. In Mansoura, Egypt, even higher prevalence values were reported, with 40.8% (49/120) positive raw meat products and 29.2% (38/130) positive ready-to-eat meat products, including beef sandwiches and burgers, with individual product prevalence ranging from 13.3% to 60% [160]. With 20% of samples testing positive, ready-to-eat meat sandwiches were also identified as a common source of contamination in Iran [156].

Overall, these findings suggest that meat products, particularly raw or undercooked meat, may occasionally harbour *H. pylori*. However, the role of meat as a significant transmission route is only weakly supported, as current data suggest that contamination is likely to be environmental or related to handling rather than originating from the animals themselves. Moreover, viability and infectious potential of *H. pylori* detected by its DNA remains questionable. Hence, current evidence indicates that meat may not be excluded as a possible but secondary transmission route.

### 4.3. Fish and Seafood

Only few studies have examined the presence of *H. pylori* in fish and seafood. As already mentioned, a study from the United States using a multiplex PCR assay reported the presence of *H. pylori* DNA in 44% of 18 tuna samples taken from ready-to-eat sushi at two restaurants in the Chicago area [162]. In Egypt, *H. pylori* was initially isolated from 9 of 115 (7.8%) *Tilapia* fish samples, but PCR amplification of the *ureC* gene confirmed only five isolates (4.4%) as *H. pylori* [163]. A Spanish study using *vacA*-specific qPCR detected *H. pylori* in 12 of 100 shellfish samples, including mussels (67%), clams (25%), and cockles (8%) [164]. Survival experiments in artificially contaminated *Mytilus galloprovincialis* demonstrated that *H. pylori* can persist for several days. Detection of *vacA* mRNA by reverse transcriptase-PCR (RT-PCR) was applied as a marker of bacterial viability and revealed survival for two days in a culturable form and four days in a VBNC state [82]. The investigation of 315 fecal samples from Egyptian fish revealed a prevalence for *H. pylori* of 6.7% by a lateral flow assay and 1.9% by PCR, with positive results limited to *Tilapia* fish (10.9% and 3.1%, respectively) [165].

Collectively, the available data indicate that fish and seafood can occasionally harbour *H. pylori* DNA, with culture confirmation being infrequent. Experimental findings support short-term survival under aquatic or refrigerated conditions. However, the lack of consistent culture-based recovery, the absence of epidemiological links or outbreak reports, and the dominance of alternative transmission pathways (oral–oral and fecal–oral) suggest that fish and seafood represent only a minor and context-dependent risk factor for *H. pylori* transmission to humans.

### 4.4. Ready-to-Eat Food

Ready-to-eat (RTE) foods, which undergo no further heat treatment prior to consumption, are particularly susceptible to contamination by pathogenic and spoilage microorganisms. Survival experiments demonstrated survival and persistence of *H. pylori* in tofu for up to five days and in leaf lettuce for two days [65]. The presence of *H. pylori* in RTE products was reported by recent studies from the Czech Republic and Iran. PCR detection of the *H. pylori glmM* gene in composite foods, such as sandwiches, baguettes, tortillas, and buns available in the Czech market network, revealed 58.6% positive samples. The analysis of other RTE products detected *H. pylori* DNA in 50% of smoked and marinated fish and 31% of salads [166]. A culture-based survey of 550 RTE food products from Iran reported isolation of *H. pylori* in 13.5% of samples, with olive salad (36%), restaurant salad (30%), fruit salad (28%), and soup (22%) most frequently contaminated [167]. Also identified as a common source of contamination in Iran were ready-to-eat meat sandwiches, with 20% of samples testing positive [156]. In Egypt, ready-to-eat meat products, including beef sandwiches and burgers, revealed a prevalence of 29.2% (38/130) positive samples [160]. Overall, data on *H. pylori* occurrence in RTE foods remain limited and are primarily based on PCR-based molecular detection, with few culture-confirmed isolates and no documented outbreak investigations. The detection of *H. pylori* DNA in such products likely reflects secondary contamination during food preparation, cross-contact with raw ingredients, or handling by infected food workers.

A large-scale study comparing *H. pylori* prevalence among individuals consuming food prepared under differing hygiene conditions analysed gastric biopsies from 1000 people across India. The analyses revealed *H. pylori* infection in 70.8% of individuals frequently consuming food from street vendors, compared with 60% among those consuming food prepared under hygienic conditions [168]. While the observed difference may reflect increased exposure to contaminated environments, the overall results suggest that street food itself is unlikely to act as a direct or primary vehicle for *H. pylori* transmission.

Hence, the available evidence suggests that RTE foods may serve as a potential but rather minor vehicle for *H. pylori* transmission, in particular under inadequate hygiene conditions.

### 4.5. Vegetables

Vegetables and salads have been investigated as potential vehicles for *H. pylori* by only few studies. A study on vegetables and salads from Iran reported isolation of *H. pylori* from 44 of 460 samples (9.6%), while PCR detection indicated a slightly higher prevalence of 10.9% [169]. In another investigation from Iran, *H. pylori* was isolated from 14% of salad samples and 13.7% of vegetable samples, with leek, lettuce, and cabbage being the most frequently contaminated (30%) [170]. Similarly, in Spain, *H. pylori* DNA was detected in 26.6% of lettuce, 9.5% of spinach, and 8.8% of chard samples [171], and a Colombian study reported detection in 25% of market lettuce and 11–25% of strawberries [172]. In contrast, studies from the Netherlands failed to detect *H. pylori* in lettuce using either culture or molecular methods [126].

Experimental survival studies have demonstrated survival of *H. pylori* for 2–4 days on various vegetables [65,76,81], with up to 6 days in a viable but non-culturable state reported for spinach [79]. Interestingly, biofilm formation appears to further enhance survival of *H. pylori* on plant surfaces, as strains exhibiting strong biofilm-forming capacity remained viable for longer periods compared to weak biofilm producers [81].

Taken together, the current findings suggest that vegetables may serve as potential vehicles for *H*. *pylori*, particularly due to their potential contact with polluted water, soil and feces. However, the inconsistent culture-based evidence, lack of outbreak data, and absence of robust epidemiological links imply that contaminated vegetables may only represent a secondary and context-dependent transmission route, particularly in regions with suboptimal sanitation.

**Table 3:** Studies on the association of *Helicobacter pylori* with food and animals.

Year	Sample Type	Country	Method	Result	Reference
1992	Blood and gastric brushings from pigs, rabbits and cows	Italy	ELISA	*H. pylori* identified in gastric brushings by a monoclonal antibody in 8 out of 10 pigs and 7 out of 10 rabbits. Raised serum IgG levels were found in 93% of pigs and 87% of rabbits.	Vaira et al. [173]
1993	Human blood	Chile	ELISA	Seropositivity of *H. pylori* antibodies in 1815 Chileans was >60% in lower socioeconomic groups and correlated with increased age, low socioeconomic status, and consumption of uncooked vegetables.	Hopkins et al. [174]
1996	Children 2–9 years old	Colombia	13C-urea breath test	*H. pylori* prevalence was overall 69%, and increased from 53% in 2-year-olds to 87% in 9-year-old children. Children who frequently consumed raw vegetables were more likely to be infected. Children in contact with sheep had increased prevalence odds.	Goodman et al. [175]
1997	Housefly	USA	Culture	*H. pylori* detected on bodies of houseflies and in their intestinal tracts. Proven dissemination of viable *H. pylori* in excreta of houseflies.	Grubel et al. [176]
1999	Gastric mucosa of horses, calves, pigs, rabbits, and chickens	Greece	Culture	*H. pylori* detected in all large-sized animals, while no positive cases observed in rabbits and chickens.	Dimola et al. [177]
1999	Raw sheep milk	Italy	PCR	*H. pylori* 16S rRNA gene detected in 60% of 51 raw milk samples from 12 sheep and *vacA* gene amplified from 5 milk samples.	Dore et al. [130]
2000	Housefly	USA	PCR	*H. pylori ureA* gene amplified in 6.9% of 3-fly or 5-fly pools of 2229 wild houseflies from several agricultural sites.	Li et al. [178]
2000	Cattle and retail beef products	USA	Culture	*Helicobacter* spp. not isolated from mucosal samples from rumen and abomasum of 105 cattle. All 20 retail beef cuts negative for *H*. *pylori*.	Stevenson et al. [25]
2001	Sheep milk and gastric tissue	Italy	Culture, PCR	*H. pylori* cultured from sheep milk and tissue samples *Helicobacter* DNA detected in 60% of 63 milk samples and 30% of 20 sheep tissue samples. The *vacA* gene of *H. pylori* amplified in 5 of 38 positive milk samples and 2 of 6 positive sheep tissue samples.	Dore et al. [131]
2002	Raw and pasteurized cow milk	Japan	Culture, PCR, EM	*H. pylori* detected by semi-nested PCR amplification of the *ureA* gene in 13 of 18 (72.2%) raw milk samples and in 11 of 20 (55%) commercial pasteurized milk samples. Culture positive for one raw milk sample but none of the pasteurized milk samples.	Fujimura et al. [132]
2002	Raw bovine milk	USA	Culture, PCR	*H. pylori* not detected either by PCR or by culture in 120 raw bovine milk samples.	Jiang and Doyle [24]
2002	Raw sheep milk	Turkey	Culture	*H. pylori* not detected in any of 440 examined raw sheep milk samples.	Turutoglu et al. [134]
2008	Raw chicken and ready-to-eat tuna	USA	Multiplex PCR	*H. pylori* DNA detected in 36% (4/11) of the raw chickens and 44% (8/18) of ready-to-eat raw tuna meat tested by a multiplex PCR assay.	Meng et al. [162]
2008	Raw goat, sheep, and cow milk	Italy	Nested PCR followed by culture	*H. pylori-*specific *glmM* gene was detected in 139 (34.7%) of 400 raw milk samples, but no isolates were obtained from the PCR-positive samples.	Quaglia et al. [133]
2011	Raw bovine milk	Greece	FISH	*H. pylori* were detected by FISH in four out of twenty (20%) raw milk samples.	Angelidis et al. [151]
2011	Serum, raw milk and feces of cows	Iran	PCR, ELISA	*H. pylori* antibodies detected in 25 (27%) of 92 cow serum specimens. Of these 25 seropositive cows, 10 (40%) fecal samples and 4 (16%) milk samples were antigen positive for *H. pylori*. Four antigen-positive milk samples revealed a positive *H. pylori* stool antigen test.	Safaei et al. [145]
2012	Raw milk from bovine, buffalo, camel, ovine, and caprine	Iran	Culture, PCR	*H. pylori* cultured from two sheep and one buffalo milk sample of 447 milk samples from 230 dairy bovine, buffalo, camel, ovine, and caprine. *H. pylori ureC* gene detected in 56 (12.5%) of milk samples, including 19 cow (14.1%), 11 sheep (12.2%), nine goat (8.7%), two camel (3.6%), and 15 buffalo (23.4%) samples.	Rahimi et al. [137]
2014	Vegetables and salads	Iran	Culture, PCR	*H. pylori* cultured from 44 out of 460 samples (9.6%). *H. pylori* DNA detected in 50 out of 460 samples (10.9%).	Atapoor et al. [169]
2014	Gastric biopsies of cow, sheep, goat, and humans	Iran	Culture, PCR	*H. pylori* detected in 164 (82%) human, 32 (16%) sheep, and 6 (3%) cow samples out of 800 gastric biopsy samples of cows, sheep, goats and humans.	Momtaz et al. [179]
2014	Raw milk and traditional dairy products	Iran	Culture, PCR	*H. pylori* detected in 103 out of 520 (19.8%) milk samples and 77 out of 400 (19.2%) traditional dairy product samples. The most frequently contaminated samples were ovine milk (35%) and traditional cheese (30%).	Mousavi et al. [138]
2014	Washed and unwashed vegetables and salads	Iran	Culture, PCR	*H. pylori* cultured from 7 out of 50 (14%) salad and 52 out of 380 (13.68%) vegetable samples. Leek, lettuce, and cabbage were the most common contaminated samples.	Yahaghi et al. [170]
2015	Fecal samples from different fish species and fish handlers	Egypt	Lateral Flow Assay (LFA), PCR	The overall prevalence rates for *H. pylori* in 315 examined fish were 6.7% for LFA and 1.9% for PCR. Only tilapia fish showed positive results by both methods (10.9% and 3.1%). 61.1% of stool samples from 18 fish handlers were positive for LFA.	Abdel-Moein et al. [165]
2015	Raw bulk tank milk of dairy cattle	Italy	Culture, PCR	Three out of 163 bulk milk samples positive for Helicobacteraceae, but not in subsequent *H. pylori*-specific PCR. *H. pylori* was not isolated.	Bianchini et al. [135]
2015	Raw bovine milk and traditional dairy products	Iran	Culture, PCR	*H. pylori* isolated from 33 (13.8%) of 120 bovine milk and 120 dairy product samples. All isolates confirmed by *ureC* PCR.	Esmaeiligoudarzi et al. [139]
2015	Raw bovine milk	Sudan	Culture, PCR	*H. pylori glmM* gene detected in 11 out of 50 (22%) raw milk samples.	Osman et al. [146]
2015	Raw milk from cow, sheep, goat, camel, buffalo; human gastric biopsies	Iran	PCR	*H. pylori* DNA detected in 12 (16%) cows, 8 (13.8%) sheep, 2 (4.8%) goats, 2 (13.3%) camels, and 4 (20%) buffalo out of 210 raw milk samples. *H. pylori* DNA detected in 82 out of 100 (82%) human samples.	Talaei et al. [143]
2016	Raw bovine milk, human stool and serum samples	Egypt	Culture, PCR, ELISA	*H. pylori glmM* gene detected in 11% of 100 raw cow milk samples. *H. pylori* antigen detected in 59% of 100 human stool samples and antibodies were detected in 50% of 100 serum samples.	Abdel-Latif et al. [147]
2016	Meat and meat products	Egypt	Culture	*H. pylori* detected in one sample of each of 30 raw meat, 20 raw poultry meat, and 20 luncheon meat samples.	El Dairouty et al. [154]
2016	Fish, ham, chicken, vegetables, meat sandwiches, minced meat	Iran	Culture, PCR	*H. pylori* isolated from 60 out of 300 (20%) food samples. Vegetable sandwich (45%), minced meat (32%) and meat sandwich (20%) positives.	Ghorbani et al. [156]
2016	Ready-to-eat food	Iran	Culture, PCR	*H. pylori* contained in 74 out of 550 (13.5%) ready-to-eat food samples. Olive salad (36%), restaurant salad (30%), fruit salad (28%) and soup (22%).	Hemmatinezhad et al. [167]
2016	Milk and meat from cow, sheep, goat, camel, and buffalo	Iran	Culture, PCR	*H. pylori* present in 92 out of 420 (21.9%) raw milk samples and in 105 out of 400 (26.2%) meat samples collected from various parts of Iran.	Saeidi and Sheikhshahrokh [142]
2017	Minced meat and hamburger samples	Iran	Culture, PCR	*H. pylori* was detected in 11 out of 150 (7.3%) meat samples with a prevalence of 1.4% for hamburger and 12.5% for minced-meat.	Gilani et al. [157]
2017	Various types of meat	Iran	Culture, PCR	*H. pylori* was detected in 11 out of 220 (5.0%) meat samples with a prevalence of 72.2% for meat samples of slaughterhouses and 27.7% for meat samples of butcheries.	Gilani et al. [158]
2017	Raw milk and dairy products	Iran	Culture, PCR	*H. pylori* was harboured by 60 out of 300 (20%) samples with a prevalence of 38.7% for raw milk and 13.2% for traditional dairy products. Ovine milk (45%) and traditional cheese (40%) revealed the highest prevalence for *H. pylori*.	Khaji et al. [140]
2017	Raw milk, meat and vegetables	Iran	Culture, PCR	*H. pylori* isolated from 40 out of 340 (11.8%) samples with a prevalence of 7.3% in meat, 16% in milk, and 12.5% in vegetable samples. Ovine milk (26%) was the most often contaminated product.	Talimkhani et al. [144]
2018	Raw milk and feces from cows, buffaloes, and sheep	Egypt	Culture, Nested PCR	*H. pylori* present in 29% percent of feces and milk samples collected from apparently healthy cows, buffaloes, and sheep examined.	Elhariri et al. [148]
2018	Raw milk, blood, and feces from cows	Algeria	Culture, PCR, ELISA	No *H. pylori* isolates obtained from milk and feces of 200 cows, while ELISA detected IgG antibodies in 12% of blood samples and 4% of milk samples from 200 cows. PCR for *glmM* gene was positive in 13% of 200 cows milk samples and confirmed all IgG-positive milk samples.	Guessoum et al. [150]
2018	Chicken meat, liver, and gizzard	Egypt	Culture, PCR	*H. pylori* isolated from 7 out of 90 (7.78%) chicken samples: 3 from chicken liver, 2 from meat, and 2 from gizzard.	Hamada et al. [159]
2018	Raw milk from bovine, ovine, caprine, buffalo, and camel	Iran	Culture, PCR	*H. pylori* cultured from 67 (10.6%) out of 630 raw milk samples. The prevalence was 17.3% in 110 samples from ovine, 13.8% in 130 caprine samples, 10.8% in 130 buffalo samples, 7.5% in 120 bovine samples, and 5.0% in 140 camel samples.	Ranjbar et al. [141]
2019	Shellfish: mussels, clams, cockles	Spain	*vacA* gene qPCR	*H. pylori vacA* gene qPCR positive for 12 out of 100 investigated samples, with 67% (8/12) positive mussels, 25% (3/12) clams, and only 8% (1/12) positive cockles.	Pina-Perez et al. [164]
2020	Feces, blood, and wool of sheep, lamb, and sheep dog	Italy	Culture, PCR, ELISA, stool antigen test (HpSA)	58 animals studied (44 sheep, 8 lambs and 6 sheep dogs). *H. pylori* antigen test positive in 82% (36/44) sheep samples and in 100% of lamb and sheep-dog stool samples. High anti- *H. pylori* IgG serum levels detected in all 6 sheep-dog and in 42 out of 44 sheep. *H. pylori* was not detected in sheep wool samples.	Dore et al. [180]
2020	Raw meat	Iran	Culture, PCR	*H. pylori* isolated from 52 out of 600 (8.66%) raw meat samples with raw ovine meat (13.07%) showing the highest prevalence.	Mashak et al. [161]
2020	Lettuce and free-living amoebae (FLA)	Nether-lands	Culture, PMA-qPCR, DVC-FISH	*H. pylori* not detected in any lettuce sample by molecular techniques or culture. Intra-amoebic *H. pylori* DNA detected by PMA-qPCR in 55% of the samples and viable intra-amoebic *H. pylori* cells in 25% of the samples by DVC-FISH.	Moreno-Mesonero et al. [126]
2021	Abomasum samples	Iraq	16S rRNA gene PCR	*H. pylori* 16S rRNA gene detected by PCR in 31 out of 150 gastric samples from sheep.	Kareem and Al-Maaly [181]
2021	Gastric biopsies of wild boar	Portugal	PCR	*H. pylori* DNA detected in 2 animals out of 14 wild boar and *H. suis* DNA detected in one animal.	Nunes et al. [182]
2022	Raw milk from cows	Egypt	Culture, PCR	*H. pylori* isolated from 13.3% out of 30 samples of cows’ milk and 6.7% out of 30 samples of marketable raw milk.	Al Sherief and Thabet [83]
2022	Chicken meat	Yemen	Culture	*H. pylori* isolated from 13.8% of 260 chicken meat samples.	Almashhadany et al. [152]
2022	Feces from goat	China	Metagenomic sequencing	*H. pylori* identified in fresh fecal samples from diarrhetic goats but not in fresh fecal samples from clinically healthy goats.	Cheng et al. [183]
2022	Chicken breast, liver, gizzard; swab samples from processing surfaces	Egypt	16S rRNA gene PCR	*H. pylori* DNA detected in 16 of 300 (5.3%) chicken samples and *H. pullorum* in 14 (4.7%) samples. Two *H. pylori* isolates were isolated from 30 swab samples from two different cutting boards.	Elrais et al. [155]
2022	Raw milk from cows and goat	Czech Republic	Culture, Nested PCR	*H. pylori* detected by nested PCR in 31 samples (40%) of 77 raw cow milk samples and 30 samples (58%) of 52 raw goats’ milk.	Furmancíková et al. [136]
2022	Raw vegetables	Spain	qPCR	*H. pylori* DNA detected in 17 (17%) out of 100 vegetable samples. 12/45 (26.6%) lettuce, 2/21 (9.5%) spinach, and 3/34 (8.8%) chard samples positive.	García-Ferrús et al. [171]
2023	Raw poultry meat	Iran	Culture, Multiplex PCR	*H. pylori* isolated from 20 of 320 (6.3%) raw chicken meat samples. Highest incidence was found in chicken raw meat (15%). No isolate was recovered from goose or quail.	Asadi et al. [153]
2023	Raw and ready-to-eat meat	Egypt	Culture, PCR	*H. pylori* detected in 40.8% (49/120) of raw meat products and in 29.2% (38/130) of ready-to-eat meat products, e.g., in 53.3% (32/60) raw ground beef, 56.7% (17/30) beef burger, 40% (8/20) beef burger sandwiches, 55% (11/20) beef shawarma sandwiches, 60% (12/20) beef kofta sandwiches, 13.3% (4/30) beef luncheon, and 15% (3/20) beef sausage sandwiches. Of 204 biochemically identified *H. pylori* isolates, 53.9% (110/204) were confirmed by PCR	Maghrabia et al. [160]
2023	Fish (*Tilapia*); water; stool samples from handlers	Egypt	Culture, PCR, stool antigen test (HpSA)	*H. pylori* isolated from 9 out of 115 (7.8%) *Tilapia* fish, but *ureC* gene PCR confirmed only five isolates (4.4%) as *H. pylori.* Culture was positive for 7 out of 50 freshwater samples, with 6 confirmed by PCR. Stool antigen test was positive for 40 of 88 fish-handlers, but culture was only positive for 35 samples (39.8%) and only 31 samples were confirmed by *ureC* gene amplification.	Mubarak et al. [163]
2023	Ready-to-eat food	Czech Republic	Nested PCR	*glmM* gene of *H. pylori* detected in 50% (n = 25) of all 50 samples. Composite food category (e.g., sandwiches, baguettes, tortillas, buns, etc.) showed highest prevalence, 58.6% (n = 21), followed by “other” (smoked and marinated fish) 50% (n = 4), and “salads” 30.8% (n = 4).	Navrátilová et al. [166]
2023	Feces from camel, sheep, and humans	Egypt	PCR, stool antigen test (HpSA)	Prevalence of *H. pylori* in 250 stool samples from sheep, camels and humans was 27.6% determined by HpSA and 24.4% determined by 16S rRNA PCR. In detail HpSA test was positive in 12% of sheep and 26% of camel stool samples. Prevalence of *H. pylori* in human stool samples was 44% determined by HpSA.	Rabah et al. [184]
2023	Feces from cattle, buffalo, sheep, dog, cat, and humans; animal milk samples	Egypt	PCR, stool antigen test (HpSA), rapid antibody test	Prevalence of *H. pylori* infection in 143 animal samples was 22.2% determined by rapid antibody test and 16% determined by HpSA. Detection rates were 28% from 50 cats, 24% from 50 dogs, 12% from 50 buffaloes, 10% from 50 sheep, and 4.7% from 43 cattle. Prevalence of *H*. *pylori* in 768 fresh human stool samples was 74.8% determined by HpSA. *H. pylori glmM* gene was detected by PCR in 21 of 27 human antigen-positive samples and 5 of 13 animal milk samples.	Shaaban et al. [149]
2024	Lettuce and strawberries	Colombia	PCR	*H. pylori* DNA detected in samples of strawberries from farms (25%) and supermarkets (11.1%), and in lettuce from markets (25%).	Vesga et al. [172]
2025	Abomasum of dairy cattle and sheep	Italy	PCR	*Helicobacteraceae-*specific DNA detected in 9% of bovine and 42% of ovine abomasa. No samples tested positive for *H. pylori.*	Recchia et al. [185]

16S rRNA: 16S ribosomal Ribonucleic Acid. ELISA: enzyme-linked immunosorbent assay; EM: electron microscopy; DVC-FISH: direct viable count fluorescence *in situ* hybridization; DWTP: drinking water treatment plants; HpSA: *Helicobacter pylori* stool antigen; LFA: Lateral Flow Assay; PCR: polymerase chain reaction; PMA-qPCR: qPCR: quantitative polymerase chain reaction; PMA-qPCR: propidium monoazide quantitative polymerase chain reaction; RT-PCR: reverse transcription polymerase chain reaction.

## 5. The Role of Animals in the Transmission of *Helicobacter pylori*

The detection of *H. pylori* in milk [132,137,138,146,150,151], meat [152,153,158] and other animal-derived products [149,177], supports the hypothesis that food may play a role in transmission, with animals serving as potential reservoirs or intermediate hosts [145].

Although *H. pylori* is a highly specialized bacterium that has evolved to colonize the human gastric mucosa, its primary and most suitable ecological niche [186], its frequent detection in milk, faeces, and internal organs of animals (Table 3) suggests possible adaptation to secondary hosts and raises questions about zoonotic transmission [187].

Genotypic analyses have revealed identical *H. pylori* strains in dogs and their owners [188], and occasional isolation from domestic cats has further reinforced the hypothesis of animal-to-human transmission [189]. However, epidemiological evidence does not consistently support this view. In a UK study involving 447 factory workers, no significant association between *H*. *pylori* seropositivity and childhood cat ownership was observed [190], while an investigation among German schoolchildren similarly reported lack of a correlation between infection and pet exposure [191]. Moreover, although *H. pylori* has been identified in non-human primates such as macaques, direct human contact with such animals is rare, rendering them unlikely to serve as significant reservoirs for human infection [9]. Recent reviews concluded that while pets, particularly dogs and cats, may transiently carry *H. pylori* or related *Helicobacter* species, their role as true zoonotic reservoirs remains unsubstantiated and likely minimal [187].

In contrast to companion animals, farm animals may act as potential vectors or transient hosts for *H. pylori*, particularly through exposure to contaminated environments or water sources [187]. Epidemiological evidence indicates that individuals in close contact with livestock are at higher risk of infection, as demonstrated by shepherds who frequently interact with sheep and dogs exhibiting elevated *H. pylori* prevalence. Thus, suggesting possible animal-associated transmission [192]. Subsequent investigations confirmed high fecal antigen positivity and elevated anti-*H. pylori* IgG titers in sheep and sheepdogs, further supporting potential exposure among these species [180].

Microscopic and molecular evidence for *H. pylori*-like organisms has also been reported in pigs, rabbits, horses, calves, and chickens, with gastric colonization appearing more consistent in larger farm animals [173,177]. In Iran, serological surveys revealed 27% *H. pylori* seropositivity among cattle, while 16–40% of fecal and milk samples tested positive for *H. pylori* antigen [145]. Other studies have reported prevalence rates ranging from 3% to 16% in gastric samples from farm animals [150,179]. These results contrast with findings from Egypt, where *H. pylori* was detected in 29% of fecal and milk samples collected from apparently healthy cows, buffaloes, and sheep [148]. Other Egyptian studies reported *H. pylori* prevalence of 12% in sheep and 26% in camels based on 250 fecal samples [184], as well as detection rates of 28% in cats, 24% in dogs, 12% in buffaloes, 10% in sheep, and 4.7% in cattle among 143 animal samples analyzed [149]. A metagenomic sequencing study in goats identified *H. pylori* exclusively in fecal samples from diarrheic animals but not in clinically healthy goats, suggesting that the infection may be transient or associated with gastrointestinal disturbance [183].

The potential infection of livestock species, such as pigs, cattle, sheep, goats, and other small ruminants, holds particular relevance, as these animals are integral to the human food chain. However, current data regarding livestock as reservoirs for *H. pylori* remain contradictory. While several studies have documented the presence of *H. pylori* DNA or viable cells in farm animals [148,173,177], other investigations failed to confirm these findings, e.g., Yousseff et al. [129] were not able to detect *H. pylori*-specific DNA in samples from livestock or companion animals in Egypt. Controversial data also exist for the presence of *H. pylori*-specific DNA in abomasum samples of cows and sheep. A study from Northern Italy detected DNA from *Helicobacter* species other than *H. pylori* in abomasum samples from dairy cows and sheep [185], while another study detected *H. pylori*-specific 16S rRNA genes in 25% of abomasum samples from 150 sheep [181]. These results very likely indicate a possible methodological variability, suggesting again that 16S rRNA gene amplification may lack specificity for *H. pylori*. The detection of the *H. pylori ureAB* gene in 2 out of 14 free-range wild boars (*Sus scrofa*) from Portugal indicates that *H. pylori* is able to colonize the stomach of such animals and game may occasionally harbour the bacterium [182].

Recently, the housefly has been proposed as a potential reservoir and mechanical vector for *H. pylori* transmission [64]. Field studies demonstrated that flies collected from swine facilities exhibited higher *H. pylori* carriage rates than those from poultry or dairy environments, while experimental inoculation studies confirmed that contaminated flies remained *H. pylori*-positive throughout their lifespan [176,178]. The findings also suggest that fly excreta may contribute to environmental contamination of food or surfaces, particularly under conditions of inadequate sanitation. Although direct evidence for fly-to-human transmission of *H. pylori* is lacking, the hypothesis remains plausible given the established role of flies in the dissemination of other enteric pathogens [64].

Collectively, available data indicate that farm animals may act as transient vectors in contaminated environments, and that flies could facilitate indirect spread. Furthermore, the detection of *H. pylori* in aquatic environments and fish underscores the potential for environmental and foodborne transmission, particularly in endemic regions. Given the capacity of *H*. *pylori* to colonize both human and animal hosts, zoonotic transmission via food or environmental pathways cannot be excluded, though current evidence supports these routes more as secondary and context-dependent pathways.

## 6. The Impact of Detection Methods on the Evidence for *Helicobacter pylori* Transmission by Food and Water

The recovery of *H. pylori* from food and environmental samples remains technically challenging due to the organism’s fastidious growth requirements. Successful cultivation necessitates microaerophilic conditions and media supplemented with selective agents to suppress competing microbiota, which are abundant in food and water matrices [193]. Most selective media were originally developed for clinical specimens and perform only suboptimal when applied to environmental or food samples [76]. Even under optimized conditions, recovery rates are consistently low [29]. Stressful environments, such as water, food matrices, or cold storage, further compromise the successful culture of *H. pylori*, resulting in underestimation of prevalence when using traditional culture-based approaches. The absence of standardized protocols for enrichment, culture, and identification contributes to a high variability across studies and renders cross-study comparisons difficult [193].

A further obstacle is *H. pylori*’s ability to transition rapidly from a culturable spiral form to a coccoid VBNC form, which is metabolically active but cannot be recovered by conventional culture [71,86,93], thus leading to potential false-negative culture results.

Because of these limitations in the culture of *H. pylori*, most investigations rely on molecular approaches for the detection of the pathogen, particularly PCR. PCR has the advantage of detecting DNA from both spiral and coccoid VBNC forms [103]. However, a key limitation is that PCR cannot discriminate between DNA from viable and dead cells. Consequently, false positive results may arise from residual DNA of non-viable bacteria. This discrepancy is evident in multiple studies where *H. pylori*-specific DNA was detected by PCR, while viable bacteria could not be isolated from the same samples [90,122,133,135,150].

The specificity of PCR-based approaches is also an issue, as 16S rRNA gene-based assays may lack the necessary specificity to discriminate *H. pylori* from other *Helicobacter* species [33], potentially inflating prevalence estimates. Variation in PCR targets across studies likely contributes to inconsistent results reported by different studies that used different targets for PCR detection of *H. pylori* in food and environmental samples [90,100,105,106]. In addition, contamination during sample collection or amplification is a particular risk with highly sensitive methods such as nested PCR, where even trace DNA from previous amplifications can generate false positives. Although contamination risks can be mitigated through strict laboratory practices (e.g., physical separation of workflows, aerosol-resistant tips, and multiple negative controls) [194], the possibility of erroneous positives remains. Conversely, false negatives may occur due to PCR inhibitors naturally present in food and environmental matrices, including fats, polysaccharides, proteins, polyphenols, humic acids, or chlorophyll [195].

To address these limitations, newer molecular approaches have been developed to assess bacterial viability. For example, methods like reverse transcriptase polymerase chain reaction (RT-PCR) targeting ribosomal RNA, direct viable count fluorescence *in situ* hybridization (DVC-FISH), or propidium monoazide quantitative Polymerase Chain Reaction (PMA-qPCR) have already been applied to detect and quantify viable *H. pylori* in water and food [89,116,196]. Comparison of DNA-based PCR versus RT-PCR for detection of *H. pylori* in water samples demonstrated that only 11 of 48 PCR-positive samples contained viable *H. pylori* [100]. Propidium monoazide qPCR (PMA-qPCR) differentiates between intact and dead bacteria by the exclusion of DNA from membrane-damaged bacteria from PCR amplification [197]. This technique has successfully confirmed the presence of viable *H. pylori* in surface water and inside free-living amoebae [98,126]. Fluorescence *in situ* hybridization (FISH) probes target ribosomal RNA as integral part of the ribosomes. Only bacteria that were viable at fixation of the cells harbour a sufficient number of ribosomes to provide a signal that is strong enough to be detected by fluorescence microscopy [198]. This approach has been successfully applied to detect viable *H. pylori* in drinking water and wastewater [85,89,95], but also detection of viable *H. pylori* in raw bulk tank bovine milk was possible [151]. Moreover, FISH demonstrated that *H. pylori* can persist in treated drinking water despite chlorination [72,116]. However, a major limitation of FISH is the low sensitivity because of the small volume of the sample that can be analysed by fluorescence microscopy, and the failure of detection of bacteria with low numbers of ribosomes [199].

Unfortunately, these advanced methods are not yet widely adopted due to their low throughput, technical complexity, and limited validation against infectivity models. Despite their drawbacks, culture methods are still applied, as they confirm viability and enable further characterization of isolates, including antimicrobial susceptibility testing, virulence profiling, and strain typing. Yet culture is constrained by low sensitivity, overgrowth by competing microbiota, and the organism’s limited survival outside its gastric niche [25,134]. As a result, many studies reporting PCR-positive findings fail to recover viable bacteria [90,122,133,135,150], leaving uncertainty over whether samples contained viable, infectious *H. pylori*. Conversely, some reports of culture-positive isolates have later been questioned due to misidentification, as illustrated by a study in Basrah, where only 10 of 173 presumptive isolates were confirmed as *H. pylori* [109].

Taken together, discrepancies between culture and PCR results underscore the methodological challenges in detecting *H. pylori* in food and environmental samples. PCR may overestimate prevalence due to DNA persistence or contamination, while culture is highly unreliable for *H*. *pylori* detection in food and environmental samples due to low sensitivity and the VBNC state of the bacteria. The most robust evidence is available from studies where isolates have been both cultured and confirmed by molecular methods [109,111,131]. Nevertheless, the possibility of sample contamination, particularly in high-prevalence settings such as rural areas of developing countries where human carriage exceeds 80% [33], must always be considered. Geographic bias also exists, as many studies reporting positive detection were conducted in Middle Eastern countries and likely reflect both higher background prevalence and methodological differences.

Although culture, PCR, and other molecular techniques have substantially advanced the detection of *H. pylori* in food and environmental matrices, each method presents inherent limitations. Interpretation of *H. pylori* positivity in such samples must therefore account for methodological constraints, the potential for cross-contamination, and the critical distinction between viable organisms and residual or non-viable DNA. This requires rigorous contamination controls, confirmation of PCR amplicons through sequencing, and incorporation of viability assays or direct culture-based isolation and confirmation approaches to verify the presence of living *H. pylori* bacteria.

To accurately elucidate the role of food and water in *H. pylori* transmission, future investigations should incorporate whole-genome sequencing (WGS) approaches. WGS is already established as a reliable method for predicting antibiotic resistance in *H. pylori* [200] and has proven invaluable for elucidating transmission dynamics in other infectious diseases [201]. Applying WGS to *H. pylori* isolates from both food-chain sources and infected individuals would enable analyses that could provide definitive evidence of strain identity and, consequently, establish direct links between environmental, foodborne, and human isolates, thereby offering the strongest possible confirmation of transmission routes for this pathogen.

## 7. Conclusions

**Figure 2:**
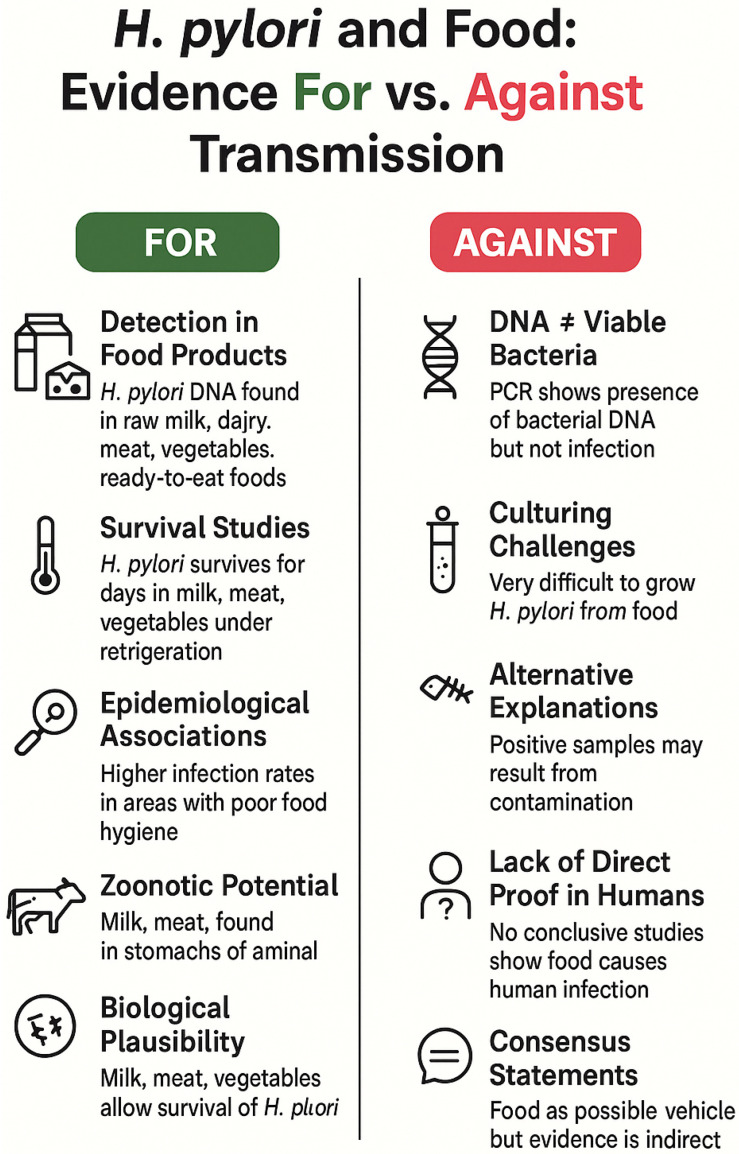
Evidence for and against food as a transmission route for *H. pylori* infection.

The possible transmission of *Helicobacter pylori* via food and water has received considerable attention, given the global burden of infection and its strong association with poor sanitation and overcrowded living conditions. Reports of *H. pylori* isolation or detection of *H. pylori*-specific DNA in milk, meat, vegetables, and drinking water provide circumstantial evidence that these matrices may serve as vehicles of exposure (Figure 2). This hypothesis is biologically plausible, as *H. pylori* can enter a VBNC state, allowing at least short-term persistence outside the host under unfavourable conditions.

Vegetables are considered of particular relevance due to their frequent contact with contaminated soil, water, or feces, particularly where hygiene during handling is inadequate, or irrigation with contaminated water is possible. Several studies have reported amplification of *H. pylori* genetic markers from lettuce, parsley, and carrots, with detection rates ranging from 10% to 40%, depending on geographic region and sampling design. For instance, a study in Chile identified *H. pylori* DNA in 27% of unwashed vegetables collected from open markets, suggesting contamination via irrigation or post-harvest handling. However, consistent with findings in milk, isolation of viable *H. pylori* from plant surfaces has rarely been achieved, likely due to the bacterium’s sensitivity to environmental stress and competition from resident microbiota.

Detection rates reported for meat, raw milk and fish show pronounced geographical variability. Most positive findings originate from developing regions, particularly in the Middle East, where *H*. *pylori* prevalence is high and hygiene standards are comparatively lower. Studies from Egypt and Iran have reported *H. pylori*-specific DNA in 20–35% of raw beef, lamb, and chicken samples using PCR-based methods. Notably, in contrast to most other food categories, several investigations have successfully cultured viable *H. pylori* from raw meat of various animal origins, including chicken, cattle, sheep, goat, camel, and buffalo. Some studies report culture-positive rates even exceeding 50% for raw beef. However, comparable data from low-prevalence, high-hygiene regions such as Europe and North America are scarce. Thus, it remains uncertain whether the elevated detection rates in the Middle East reflect genuine contamination linked to endemic infection or differences in methodology and hygiene. These discrepancies emphasize the need for standardized detection protocols and geographically comparative studies. While the recovery of viable *H. pylori* from raw meat supports its potential as a transmission vehicle, the bacterium’s inability to survive cooking makes meat an unlikely route of infection for the general population.

Collectively, the detection of *H. pylori* DNA in diverse food matrices, including milk, meat, vegetables, and RTE products, indicates widespread environmental exposure to the bacterium or its genetic remnants. However, the frequent failure to recover viable *H. pylori* underscores major methodological and biological challenges. The organism’s fastidious growth requirements, including microaerophilic conditions and complex nutrient media, hinder successful isolation from food and environmental samples. Moreover, the lack of standardized enrichment, culture, and identification protocols contributes to inconsistent results across studies, complicating direct comparison and meta-analytical assessment. This methodological variability is a central reason why the role of food and water in transmission of *H. pylori* infection remains controversial.

Accurate assessment of *H. pylori* transmission via food and water requires methodological rigor and integrated analytical approaches. Future research should apply stringent contamination controls, verify PCR products by sequencing, and incorporate viability assays such as propidium monoazide (PMA)-qPCR or mRNA detection by RT-PCR. Whenever feasible, direct culture and characterization of isolates should be performed in parallel with molecular detection and the application of whole-genome sequencing (WGS) to establish direct connections between *H. pylori* isolates obtained along the food chain and infected humans. Integrating these approaches with epidemiological and experimental evidence will be essential to determine the routes of *H. pylori* transmission and whether the pathogen can persist and remain infectious in foodborne and environmental contexts.

Overall, available evidence suggests that food and water may act as occasional or opportunistic transmission routes, particularly in settings with poor sanitation infrastructure. However, current data remain insufficient to classify them as major pathways of infection. The most robust evidence continues to support person-to-person transmission, particularly within families, as the predominant route. Oral–oral spread through saliva or shared utensils and fecal–oral transmission associated with inadequate hygiene are strongly supported by epidemiological data, including household clustering and early childhood acquisition. Food and water, while potentially contributory under specific conditions, likely represent secondary or context-dependent transmission vehicles within the broader epidemiology of *H. pylori*.

## Data Availability

No new data were created or analyzed in this study.

## Abbreviations

The following abbreviations are used in this manuscript:

ATCC	American Type Culture Collection
cagA	cytotoxin-associated gene A
CTC	Cyanoditoyl Tetrazolium Chloride
DNA	Deoxyribonucleic Acid
DOI	Digital Object Identifier
DVC	Direct Viable Count
DWTP	Drinking Water Treatment Plant
EM	Electron Microscopy
ELISA	Enzyme-Linked Immunosorbent Assay
FISH	Fluorescence *in situ* Hybridization
FLA	Free Living Amoebae
glmM	phosphoglucosamine Mutase
HpSA	*Helicobacter pylori* Stool Antigen
LFA	Lateral Flow Assay
MPN	Most Probable Number
mRNA	messenger Ribonucleic Acid
NGS	Next-Generation Sequencing
PAI	Pathogenicity Island
PCR	Polymerase Chain Reaction
PMA	Propidium Monoazide
PPI	Proton Pump Inhibitor
qPCR	quantitative Polymerase Chain Reaction
RTE	Ready-To-Eat
rRNA	ribosomal Ribonucleic Acid
RT-PCR	Reverse Transcription Polymerase Chain Reaction
T4SS	Type IV Secretion System
UHT	Ultra Heat Treated
UK	United Kingdom
US	United States
ureA	urease A
ureB	urease B
ureC	urease C
vacA	vacuolating cytotoxin A
VBNC	Viable But Nono-Culturable
WGS	Whole Genome Sequencing
